# Microhomology-mediated end-joining-dependent integration of donor DNA in cells and animals using TALENs and CRISPR/Cas9

**DOI:** 10.1038/ncomms6560

**Published:** 2014-11-20

**Authors:** Shota Nakade, Takuya Tsubota, Yuto Sakane, Satoshi Kume, Naoaki Sakamoto, Masanobu Obara, Takaaki Daimon, Hideki Sezutsu, Takashi Yamamoto, Tetsushi Sakuma, Ken-ichi T. Suzuki

**Affiliations:** 1 Department of Mathematical and Life Sciences, Graduate School of Science, Hiroshima University, 1-3-1 Kagamiyama, Higashi-Hiroshima, Hiroshima 739-8526, Japan; 2 Transgenic Silkworm Research Unit, National Institute of Agrobiological Sciences, 1-2 Owashi, Tsukuba, Ibaraki 305-8634, Japan; 3 Department of Biological Science, Graduate School of Science, Hiroshima University, 1-3-1 Kagamiyama, Higashi-Hiroshima, Hiroshima 739-8526, Japan; 4 Insect Growth Regulation Research Unit, National Institute of Agrobiological Sciences, 1-2 Owashi, Tsukuba, Ibaraki 305-8634, Japan

## Abstract

Genome engineering using programmable nucleases enables homologous recombination (HR)-mediated gene knock-in. However, the labour used to construct targeting vectors containing homology arms and difficulties in inducing HR in some cell type and organisms represent technical hurdles for the application of HR-mediated knock-in technology. Here, we introduce an alternative strategy for gene knock-in using transcription activator-like effector nucleases (TALENs) and clustered regularly interspaced short palindromic repeats (CRISPR)/CRISPR-associated 9 (Cas9) mediated by microhomology-mediated end-joining, termed the PITCh (Precise Integration into Target Chromosome) system. TALEN-mediated PITCh, termed TAL-PITCh, enables efficient integration of exogenous donor DNA in human cells and animals, including silkworms and frogs. We further demonstrate that CRISPR/Cas9-mediated PITCh, termed CRIS-PITCh, can be applied in human cells without carrying the plasmid backbone sequence. Thus, our PITCh-ing strategies will be useful for a variety of applications, not only in cultured cells, but also in various organisms, including invertebrates and vertebrates.

## Abstract (web summary)

One challenge facing the use of programmable nucleases in genome engineering is the requirement for homologous recombination. Here, Nakade *et al.* harness microhomology-mediated end-joining as a means of inserting exogenous coding sequences into the genome using both TALEN and CRISPR/Cas9 technologies.

## Graphical abstract

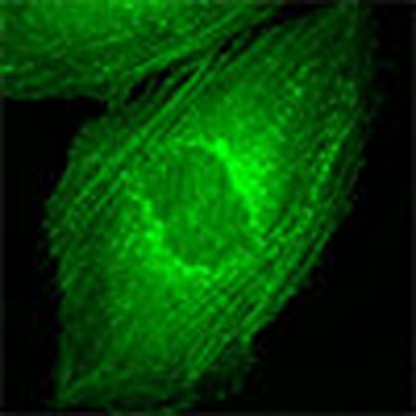

Programmable nucleases, such as transcription activator-like effector nucleases (TALENs) and RNA-guided endonucleases, that is, clustered regularly interspaced short palindromic repeats (CRISPR)/CRISPR-associated 9 (Cas9), have been used widely for genetic engineering, including gene knockout, knock-in and various chromosomal rearrangements^1^,^2^. Gene knock-in has generally been achieved by co-introduction of programmable nucleases and single-stranded oligonucleotides^3^,^4^ or a targeting vector harbouring left and right homology arms^5^,^6^, inducing homologous recombination (HR)-dependent gene addition. Although HR-mediated gene knock-in allows precise insertion of large DNA fragments, construction of targeting vectors is often laborious and targeting efficiency depends on the substantial variation in the frequency of HR induction among cell types and organism species. However, the addition of complementary overhangs to donor DNA fragments or simple linearization of donor DNA plasmids has been shown to facilitate targeted integration mediated by non-homologous end-joining both in cultured cells^7^,^8^ and in zebrafish^9^. Obligate ligation-gated recombination has also reportedly been able to integrate plasmid DNA into a targeted genomic locus^10^. These methods use programmable nucleases to make a DNA double-strand break (DSB) that leaves 5′ overhangs (zinc finger nucleases (ZFNs) and TALENs) or blunt ends (CRISPR/Cas9), and then rely on the ligation of similar ends on the chromosomal target site and the insert. These targeted integrations can thus be considered to represent ‘simple ligation’.

Conversely, microhomology-mediated end-joining (MMEJ)-dependent mutations have frequently been found in programmable nuclease-mediated gene disruption without exogenous donors^11^. MMEJ is a DSB repair mechanism that uses microhomologous sequences (5–25 bp) for error-prone end-joining^12^. In the cell cycle, MMEJ repair is active during G1/early S phases, whereas HR is active during late S/G2 phases^13^. Therefore, we devised a novel MMEJ-mediated gene knock-in strategy, referred to as the PITCh (Precise Integration into Target Chromosome) system, which enables efficient targeted integration of large DNA fragments in a wide range of cells and organisms, even those with low HR activity. We demonstrate the insertion of exogenous reporter genes into human cells and animals using the PITCh system with TALENs and CRISPR/Cas9. Our PITCh methods provide a new insight into the targeted insertion of exogenous donor DNA and an alternative way of making knocked-in cells and organisms.

## Results

### TAL-PITCh design and application in human cells

**Figure 1:**
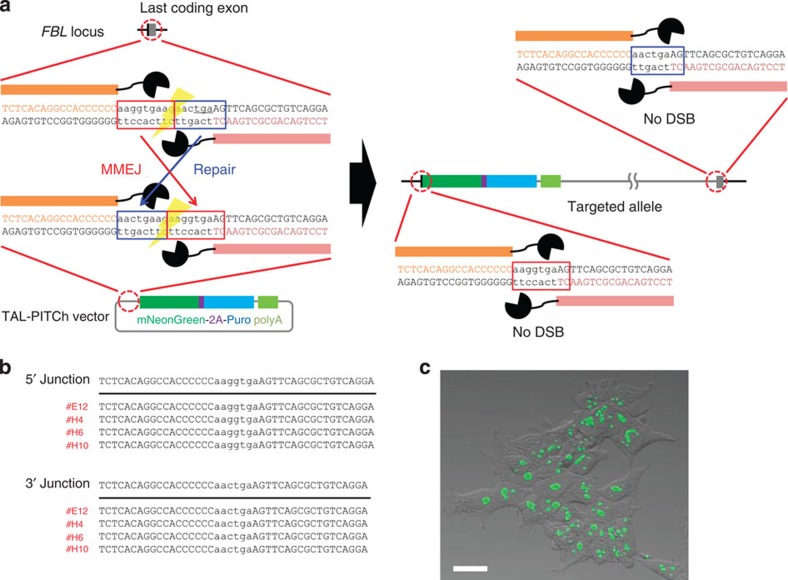
TAL-PITCh in human cells. (**a**) Schematic illustration of TAL-PITCh at the human *FBL* locus. Orange and pink letters indicate the left and right TALEN target sites, respectively. Red and blue boxes indicate the microhomologous sequences. The stop codon is underlined. (**b**) Sequences of knocked-in clones. The intended knocked-in sequence is shown at the top. TALEN target sites are shown in capital letters. Red letters indicate correctly knocked-in clones. (**c**) Confocal laser scanning microscopy image of knocked-in cells showing nucleolar localization of mNeonGreen fluorescence (clone #H6). Scale bar, 30 μm.

We first demonstrated the PITCh system in TALEN-mediated knock-in (TAL-PITCh). In TAL-PITCh, a single pair of TALENs and a TAL-PITCh vector containing a TALEN target site are constructed and co-introduced (Fig. 1a, left panel). To generate microhomologous sequences, the TALEN target site on the TAL-PITCh vector should contain a different spacer sequence compared with the original genomic sequence, in which the anterior half and posterior half are switched. The genomic sequence and the TAL-PITCh vector can be cut by the same TALEN pair, and the linearized TAL-PITCh vector contains microhomologous DNA ends corresponding to the genomic cleavage site. After MMEJ-dependent integration, the whole vector is precisely incorporated into the genome with two TALEN target sites (Fig. 1a, right panel). However, these TALEN target sites are hardly cut by TALENs, because they contain shortened spacer regions, which are out of the optimal range for DSB introduction by TALENs^14^.

As a proof-of-principle experiment, we first demonstrated the TAL-PITCh system in cultured cells. We targeted the last coding exon of the human *fibrillarin* (*FBL*) gene using Platinum TALENs^15^, and knocked-in the TAL-PITCh vector in HEK293T cells, resulting in a C-terminal fusion of *mNeonGreen*, reported recently as an ultra-bright fluorescent protein gene^16^, followed by *2A-puromycin*. The TAL-PITCh vector contains no promoter for mammalian cell expression; therefore, the *FBL-mNeonGreen-2A-puromycin* gene expression should be driven by the endogenous *FBL* promoter. After puromycin selection, single cells were isolated by limiting dilution and cultured independently. Six potential knocked-in clones were analysed by DNA sequencing and laser-scanning fluorescence microscopy. Genomic regions around the 5′ and 3′ junctions could be amplified by PCR and sequenced from four of the six clones (Supplementary Fig. 1a,b; Supplementary Table 1). All the sequenced clones had correctly targeted alleles mediated by MMEJ (Fig. 1b), and showed nucleolar fluorescence, which is consistent with a previous report^16^ (Fig. 1c).

To test the applicability of TAL-PITCh for another genomic locus and another cell line, we targeted the human *β-actin* (*ACTB*) gene in HeLa cells (Supplementary Fig. 2a). Six potentially knocked-in cell clones showing fluorescence were established and their junctions were analysed by PCR. Four of the six were selected as correctly PITChed candidate clones (Supplementary Fig. 3a,b; Supplementary Table 1). In this case, one of the four clones contained a 3-bp insertion at the 5′ junction and three of the clones contained 5–27-bp insertions and deletions at the 3′ junction; one clone had correct junctions at both sides (Supplementary Fig. 2b). Fluorescence was observed at stress fibres in this clone (Supplementary Fig. 2c). We further confirmed correct integration by southern blot analysis, indicating that no random integration occurred (Supplementary Fig. 4). We also confirmed the higher colony-forming efficiency than TALEN-assisted gene knock-in mediated by HR, suggesting the superiority of MMEJ-mediated integration compared with the conventional method (Supplementary Fig. 5).

### TAL-PITCh in animals

To check the applicability of the TAL-PITCh system *in vivo*, we next examined TAL-PITCh in silkworms (*Bombyx mori*) and frogs (*Xenopus laevis*). In silkworms, TALENs can induce highly efficient mutagenesis of the target genes, and the mutation rates in G_0_ gametes can exceed 50%^17^. Nevertheless, a successful knock-in of a long gene cassette using TALENs has not yet been achieved. This is presumably because HR activity is very low in germline cells of silkworms^17^ and suggests that conventional knock-in methods mediated by HR are not promising. Therefore, we conceived the idea of applying the MMEJ-mediated TAL-PITCh system in silkworms.

**Figure 2:**
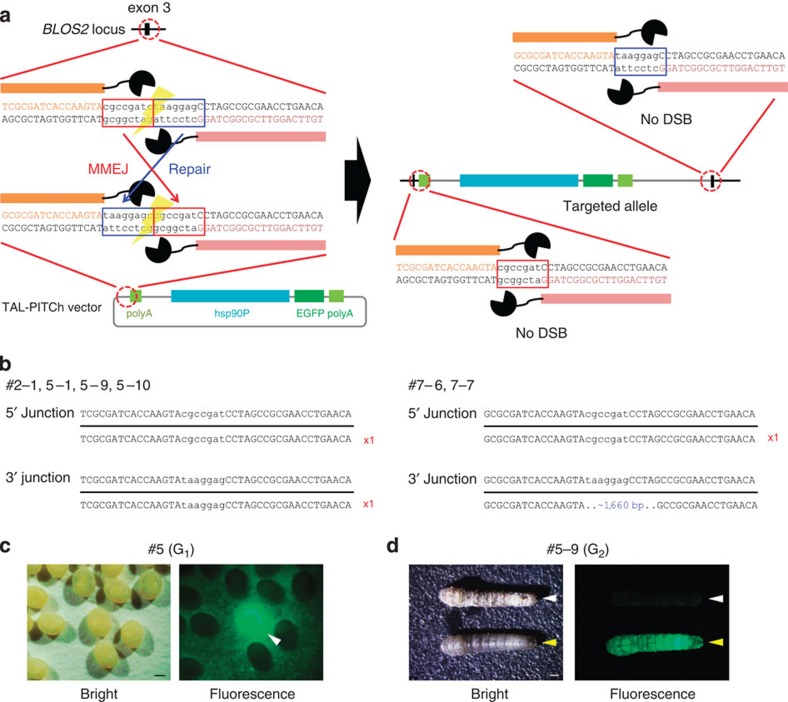
TAL-PITCh in silkworms. (**a**) Schematic illustration of TAL-PITCh at the *B. mori BLOS2* locus. Orange and pink letters indicate the left and right TALEN target sites, respectively. Red and blue boxes indicate the microhomologous sequences. hsp90P, *hsp90* promoter. (**b**) Sequences of knocked-in alleles from the six G_1_ worms (#2–1, #5–1, #5–9, #5–10, #7–6 and #7–7). The intended knocked-in sequence is shown at the top. TALEN target sites are shown in capital letters. Red letters indicate correctly knocked-in alleles. Blue letters indicate insertions. (**c**) Bright-field and fluorescence microscopy images of the G_1_ embryos in the #5 batch. Strong EGFP expression could be observed in the putative knock-in embryo, according to the activity of the *hsp90* promoter (arrowhead). Scale bar, 0.5 mm. (**d**) Bright-field and fluorescence microscopy images of the G_2_ larva derived from #5–9 batch. White and yellow arrowheads indicate wild-type and knock-in silkworms, respectively. Note that the knock-in larva shows an oily skin phenotype. Scale bar, 1 mm.

We targeted the silkworm *BLOS2* gene, because its efficient knockout has previously been achieved using TALENs^18^. Messenger RNA (mRNA) of TALENs designed against exon 3 of the *BLOS2* gene was injected together with the TAL-PITCh vector harbouring the *hsp90* promoter-enhanced green fluorescent protein (*EGFP*) expression cassette^19^ (Fig. 2a), and the EGFP expression in their progeny was examined. Remarkably, a number of G_1_ embryos showed strong EGFP expression (Fig. 2c; Supplementary Fig. 6; Supplementary Table 2). This result suggested that the knock-in had occurred successfully in the G_0_ gametes. We checked the genotype of each EGFP-positive G_1_ individual and found that the TAL-PITCh vector was integrated into the *BLOS2* locus in six worms (Fig. 2b; Supplementary Fig. 7; Supplementary Table 2). Four of them showed precise integration, whereas two of them had ~1,660-bp extra sequence containing a partial *EGFP* sequence and the genomic sequence at ~2.6-kb downstream of the TALEN target site in the 3′ junction (Fig. 2b; Supplementary Table 2). The targeted integration into the *BLOS2* locus was further supported by the fact that these individuals exhibited an oily skin, a phenotype caused by the disruption of *BLOS2* gene^18^ (Fig. 2d). Thus, we concluded that the TAL-PITCh system is quite effective in silkworms.

**Figure 3:**
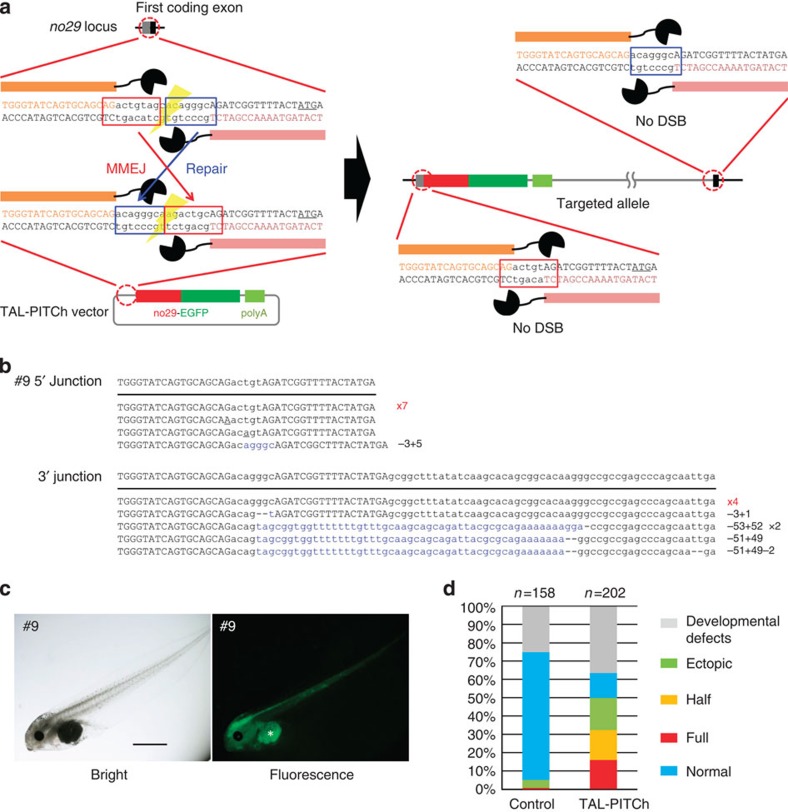
TAL-PITCh at the *no29* locus in frog embryos. (**a**) Schematic illustration of TAL-PITCh at the *X. laevis no29* locus. Orange and pink letters indicate the left and right TALEN target sites, respectively. Red and blue boxes indicate the microhomologous sequences. The start codons are underlined. (**b**) Sequences of knocked-in alleles from embryo #9. The intended knocked-in sequence is shown at the top. TALEN target sites are shown in capital letters. Red letters indicate correctly knocked-in alleles. Blue letters indicate insertions. Dashes indicate deletions. Substitutions are underlined. (**c**) Bright-field and fluorescence microscopy images of embryo #9. An asterisk indicates yolk autofluorescence. Scale bar, 1 mm. (**d**) Percentage of phenotypes in the control embryos and the TAL-PITChed embryos. For the control, the vector from which the TALEN target site was removed was used instead of the TAL-PITCh vector. Except for abnormally developed embryos, phenotypes were divided into four groups (full, half, ectopic and normal) according to the expressed region of EGFP. Total numbers of individuals are shown at the top of each graph.

We subsequently tried *EGFP* knock-in at endogenous gene loci in *X. laevis* embryos as a model of vertebrates, because gene knock-in in frogs including *X. laevis* has not yet been achieved, although targeted mutagenesis can be performed efficiently using TALENs^20^,^21^. Thus, we first targeted the *no29* locus, one of the histone chaperone paralogues^22^ in *X. laevis*, using TAL-PITCh (Fig. 3a). In this case, we designed TALENs around the start codon of the *no29* gene and knocked-in the *no29*-*EGFP* fusion complementary DNA. Although the spatial expression pattern of *no29* during early development of *X. laevis* has never been elucidated, we observed an obvious expression tendency in the central nervous system (Fig. 3c; Supplementary Fig. 8b). Overall, ~15% of the embryos injected with the TALEN mRNAs and the TAL-PITCh vector showed full expression and another 15% of the embryos showed half expression, that is, the left half or the right half of the body, in the central nervous system (Fig. 3d). Three individuals showing the intended sizes of amplicons for both the 5′ and 3′ junctions were sequenced, and all three had precisely PITChed alleles at least in the 5′ junction. In the 3′ junction, however, not all the individuals contained precisely PITChed alleles (#1, 9 and 10; Fig. 3b; Supplementary Fig. 8a,b).

**Figure 4:**
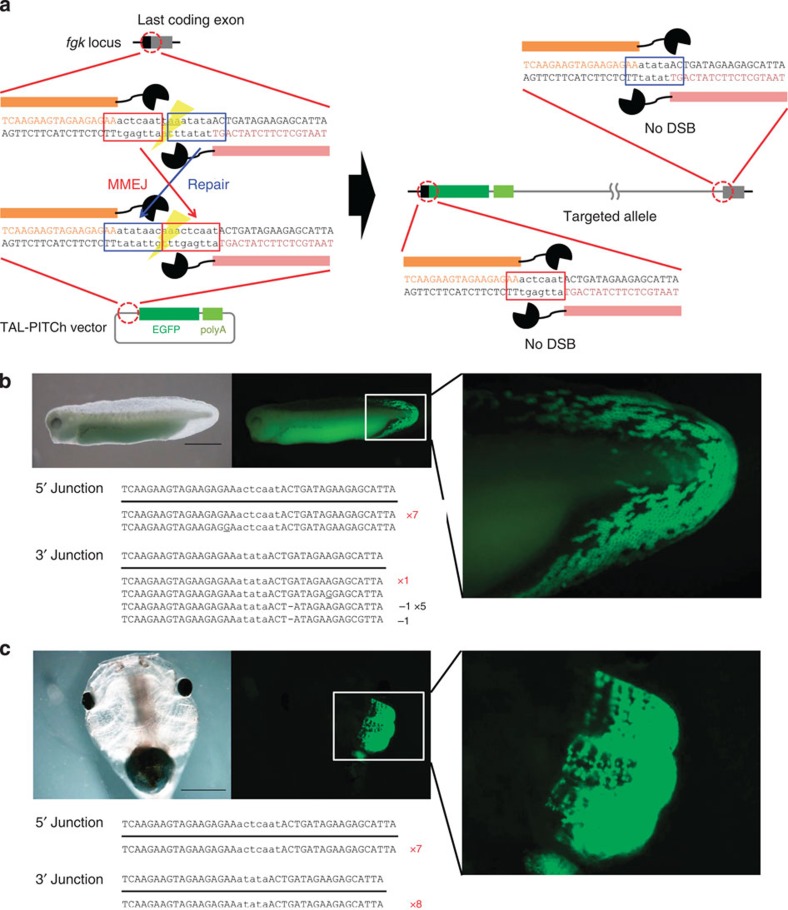
TAL-PITCh at the *fgk* locus in frog embryos. (**a**) Schematic illustration of TAL-PITCh at the *X. laevis fgk* locus. Orange and pink letters indicate the left and right TALEN target sites, respectively. Red and blue boxes indicate the microhomologous sequences. The stop codon is underlined. (**b**,**c**) Microscopic images and sequences of the TAL-PITChed embryos showing EGFP expression in the fin (**b**) and the gill (**c**). TALEN target sites are shown in capital letters. Red letters indicate correctly knocked-in alleles. Substitutions are underlined. Scale bars, 1 mm.

We next demonstrated TAL-PITCh-mediated *in vivo* gene knock-in at the *keratin* (*fgk;* fin and gill keratin) locus in *X. laevis*, in a manner similar to that in human cells (Fig. 4a). The *EGFP* gene was inserted just before the endogenous stop codon to express an *fgk*–*EGFP* fusion gene. Regarding *fgk*, transgenic *X. laevis* embryos have reportedly shown specific expression in the fin and the gill^23^. Consistent with this report, we obtained several embryos showing fluorescence specifically localized in the fin (Fig. 4b) and the gill (Fig. 4c) with precise 5′ and 3′ junctions, although one of them also contained subtle mutations at the both junctions (Fig. 4b,c). Furthermore, fusional expression enabled us to observe a cytoskeletal localization (Fig. 4b,c).

### CRIS-PITCh design and application in human cells

**Figure 5:**
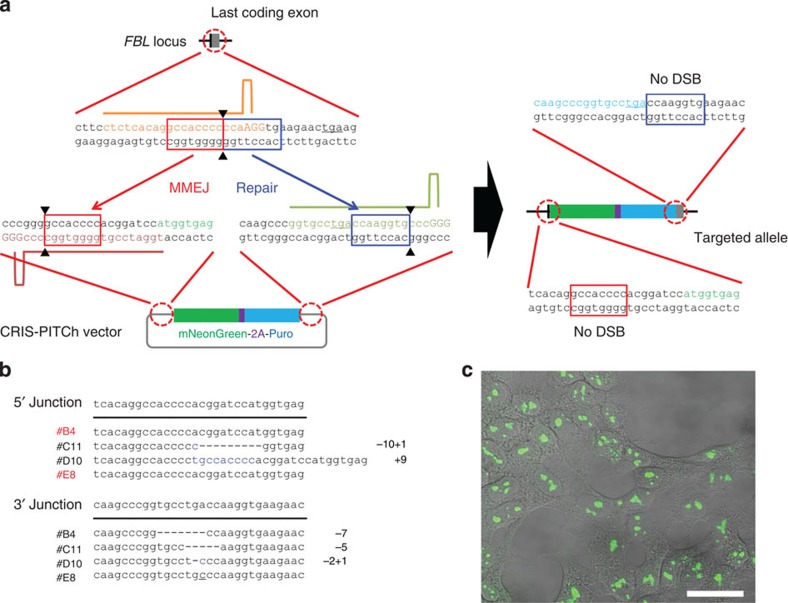
CRIS-PITCh in human cells. (**a**) Schematic illustration of CRISPR/Cas9-mediated targeted integration using CRIS-PITCh. Orange, pink and green letters indicate the gRNA target sites. Red and blue boxes indicate the microhomologous sequences. The stop codons are underlined. (**b**) Sequences of knocked-in clones. The intended knocked-in sequence is shown at the top. Dashes indicate deletions. Blue letters indicate the insertion. The substitution is underlined. (**c**) Confocal laser scanning microscopy image of knocked-in cells showing nucleolar localization of mNeonGreen fluorescence (clone #B4). Scale bar, 30 μm.

Another important facet of the PITCh system is whether CRISPR/Cas9 could be used instead of TALENs. Thus, we targeted the *FBL* locus in HEK293T cells using CRISPR/Cas9-mediated PITCh (CRIS-PITCh) (Fig. 5a, left panel). The principles of inducing DSBs with TALENs and CRISPR/Cas9 are totally different; therefore, we modified the targeting strategy (Fig. 5a). In CRIS-PITCh, three guide RNAs (gRNAs) and Cas9 nuclease should be coexpressed, and two different gRNA target sites should be added to the CRIS-PITCh vector. Strategies used for TAL-PITCh can, of course, also be applicable to CRIS-PITCh; however, there are several reasons for improving the system: to remove unnecessary vector backbone and to abolish restriction on the gRNA target sequence (see Discussion for details). Using this improved CRIS-PITCh system enabling cassette integration, we could produce knocked-in cells without any additional sequence (Fig. 5a, right panel). After transfection of CRISPR/Cas9 and CRIS-PITCh vectors, followed by puromycin selection and single-cell cloning, genomic DNA was extracted and knocked-in alleles were amplified by PCR (Supplementary Fig. 9). DNA sequencing revealed that two of the four sequenced clones contained precisely joined 5′ junctions, while they had substitutions, insertions or deletions at the 3′ junction (Fig. 5b). Nucleolar localization of fluorescence was observed, similar to the TAL-PITCh experiment (Fig. 5c).

Finally, we investigated whether the TAL-PITChed and CRIS-PITChed cell clones contained off-target mutations, especially because the CRISPR/Cas9 system can reportedly induce substantial off-target mutations in human cell lines, such as U2OS, K562 and HEK293 cells^24^,^25^,^26^. The top six potential off-target sites were sequenced for the TAL-PITChed HEK293T cell clones (#H4 and #H6 in Fig. 1b and Supplementary Fig. 1), and the top three potential off-target sites of each gRNA were sequenced for the CRIS-PITChed HEK293T cell clones (#B4 and #E8 in Fig. 5b and Supplementary Fig. 9b). To our relief, none of the sequenced off-target candidates were mutated (Supplementary Tables 3 and 4).

## Discussion

Along with the HR-independent knock-in strategies reported so far, our PITChing strategy enables flexible gene knock-in in cells and animals. In this study, we examined full-plasmid integration using TAL-PITCh and cassette integration using CRIS-PITCh. However, cassette integration using TAL-PITCh without the vector backbone and full-plasmid integration using CRIS-PITCh may also be possible. When performing cassette integration using TAL-PITCh, two TALEN target sites should be added at both ends of the cassette (Supplementary Fig. 10). The left half of the spacer sequence of the genomic TALEN target site should be placed at the right half of the spacer region of the left TALEN site on the TAL-PITCh vector, and the right half of the spacer sequence of the genomic TALEN target site should be placed at the left half of the spacer region of the right TALEN site on the vector. Alternatively, additional Cre-loxP- or Flp-FRT-mediated excision after establishing knocked-in cells or minicircle DNA generation^27^ after constructing plasmid vectors could also be performed, as discussed in the previous study^10^.

There are two ways to perform full-plasmid integration using CRIS-PITCh, and restriction of the target genomic sequence varies between them. When gRNA for the CRIS-PITCh vector is designed against the sense strand, sequence restriction would be 5′-GGNNNNNNGG-3′, if the microhomologous sequence is set to 8 bp (Supplementary Fig. 11a). However, when gRNA for the CRIS-PITCh vector is designed against the antisense strand, the sequence restriction would be 5′-CCGG-3′, if the microhomologous sequence is set to 8 bp (Supplementary Fig. 11b). Modifying the length of the microhomology sequence can change these restrictions of the target sequence.

Other tips for the TAL-PITCh and CRIS-PITCh are described below. Regarding TAL-PITCh, the left and right TALEN target sites on the TAL-PITCh can be shuffled. If they are shuffled, the 5′ junction of the targeted allele would contain the left TALEN site, a shortened spacer and an inverted left TALEN site. The 3′ junction of the targeted allele would contain the right TALEN site, a shortened spacer and the inverted right TALEN site. By performing shuffling, we can completely avoid recutting the targeted allele by TALENs, if we use TALENs containing heterodimeric FokI nuclease domains^28^,^29^,^30^. Regarding CRIS-PITCh-mediated cassette integration, the left and right gRNA target sites on the CRIS-PITCh vector should be designed to target the antisense and sense strands, respectively, as shown in Fig. 5a. Otherwise, some sequence restriction other than the original protospacer adjacent motif of the genomic CRISPR/Cas9 target site will arise. When targeting the antisense strand at the genomic CRISPR/Cas9 target site, the left and right gRNA target sites on the CRIS-PITCh vector should be designed to target the sense and antisense strands, respectively, to minimize the limitation of the genomic CRISPR/Cas9 target site.

In our TAL-PITCh and CRIS-PITCh experiments, the 5′ junctions had a high tendency to be joined precisely using the microhomologous sequence; however, the 3′ junctions were not necessarily joined by MMEJ. This tendency is likely to depend on the selection method that we adopted in this study. Puromycin selection used in this study can exclude out-of-frame clones; however, the 3′ junction is thought to have little influence on selection. In the case where both junctions need to be joined correctly for drug or fluorescence selection, it is likely that the bias observed in this study will disappear. In addition, enhancement of the MMEJ repair pathway and/or suppression of the NHEJ repair machineries might also increase the targeted integration with precise junctions and decrease the NHEJ-dependent erroneous integration, including false-positive clones, such as #F3 and #B10 in Supplementary Fig. 1b, #2 and #6 in Supplementary Fig. 3b and #G10 in Supplementary Fig. 9, which supposedly have unintended knocked-in alleles; for example, integration of concatemerized vectors, integration with large deletion or addition and random integration. Further studies are needed to clarify the mechanism of integration and improve its accuracy.

Overall, we proved that TAL-PITCh-mediated gene knock-in could be applied in human cells and other animals, suggesting broad applicability of the strategy. To the best of our knowledge, this is the first report to show that targeted insertions can occur via very short microhomologies, both in cultured cells and in animals. In addition, we demonstrated successful CRIS-PITCh-mediated gene knock-in in human cells without carrying over a vector backbone sequence. We anticipate that our PITCh systems will enhance the usefulness of genome engineering techniques in a variety of cells and organisms, especially in those in which gene knock-in is difficult because of low HR efficiency.

## Methods

### Construction of TALEN plasmids

For human cell and frog experiments, a two-step Golden Gate assembly method using the Platinum Gate TALEN Kit (Addgene; cat#1000000043)^15^ was used to construct Platinum TALEN plasmids containing the homodimer-type FokI nuclease domain. Briefly, single DNA-binding repeats were assembled into the intermediate array vectors. The assembled repeat arrays were subsequently inserted into the final destination vectors, ptCMV-153/47-VR. For the silkworm experiments, the Golden Gate TALEN and TAL Effector Kit 2.0 (Addgene; cat#1000000024)^31^ were used to construct TALENs (BLTS-5A and BLTS-4B^18^), and the repeat arrays were inserted into the scaffold plasmid, pBlue-TAL^18^.

### Construction of CRISPR/Cas9 plasmids

The multiplex CRISPR/Cas9 assembly system^32^ was used to construct the all-in-one CRISPR/Cas9 plasmids. Briefly, pX330 vector (Addgene; Plasmid 42230) was modified to unify multiple gRNA-expressing cassettes into a single vector using the Golden Gate assembly method. Oligonucleotides for gRNA templates were synthesized, annealed and inserted into the corresponding vectors. A list of the oligonucleotides used is shown in Supplementary Table 5. Golden Gate assembly was used to assemble the constructed vectors into an all-in-one CRISPR/Cas9 vector for the *FBL* gene, termed pX330A-*FBL*-3gRNAs, harbouring three gRNA cassettes and a Cas9 cassette.

### Construction of PITCh and HR vectors

TAL-PITCh, CRIS-PITCh and HR vectors were constructed using PCR and In-Fusion cloning (Clontech) or by standard molecular-cloning methods. The full-plasmid sequences are shown in Supplementary Fig. 12.

### Cell culture and transfection

HEK293T and HeLa cells, obtained from ATCC, were maintained in Dulbecco’s modified Eagle’s medium supplemented with 10% fetal bovine serum. Lipofectamine LTX (Life Technologies) and Opti-MEM (Life Technologies) were used to transfect plasmids, according to the supplier’s protocols. Plasmid concentrations, cell numbers and dishes used were as follows: 200 ng each for ptCMV left and right TALEN vectors and for TAL-PITCh vector into 1 × 10^5^ cells using a six-well plate in the experiments in Fig. 1 and Supplementary Fig. 2; 1.6 μg each for ptCMV left and right TALEN vectors and for TAL-PITCh or HR vector into 5 × 10^5^ cells using a 100-mm dish in the experiments of Supplementary Fig. 5; 400 ng for pX330A-*FBL*-3gRNAs CRISPR/Cas9 vector and 200 ng for CRIS-PITCh vector into 1 × 10^5^ cells using a six-well plate in the experiments of Fig. 5. After transfection, cells were cultured in the growth medium described above for 3 days and then selected with 1 μg ml^−1^ puromycin for 6 days. For DNA sequencing, microscopy and southern blotting, the selected cells were cloned using the limiting dilution method in 96-well plates.

### mRNA synthesis and microinjection for the silkworm experiments

mMessage mMachine T7 Ultra Kit (Life Technologies) was used to synthesize *B. mori BLOS2* TALEN mRNA (BLTS-5A and BLTS-4B^18^). mRNA was precipitated with LiCl, washed with 70% ethanol three times and air-dried and dissolved in 0.5 mM phosphate buffer (pH 7.0) containing 5 mM KCl. TALEN mRNA (250 ng μl^−1^ each) and 500 ng μl^−1^ donor vector were injected into embryos of silkworm *w1*-*pnd* strain that were collected between 1- and 2 h after egg laying at the syncytial preblastderm stage. After injection, the opening was sealed with glue and the embryos were incubated at 25 °C. The hatched silkworm larvae were reared on an artificial diet (Nihon Nosan Kogyo) at 25 °C under a 12-h light/dark photoperiod. Each injected individual was crossed with non-injected worms. EGFP expressions of G_1_ individuals were observed during the embryonic stage, and the EGFP-positive embryos were selected for further rearing. The G_1_ adults were crossed with the *w-c* diapausing strain.

### mRNA synthesis and microinjection for the frog experiments

Fertilized *X. laevis* eggs were obtained from wild-type adults injected with human chorionic gonadotropin (Aska Pharmaceutical). Eggs were dejellied with 2% cysteine and washed in 0.1 × Marc’s modified ringer (MMR). Washed eggs were transferred into 5% Ficoll (Sigma-Aldrich) in 0.3 × MMR and were co-injected with a pair of TALEN mRNAs (250 pg each), synthesized using a mMessage mMachine T7 Ultra Kit (Life Technologies), and TAL-PITCh vectors (100 pg) at the one-cell stage using Nanoject II (Drummond). Injected embryos were reared to the swimming stage in 0.1 × MMR at 20 °C. Animals were maintained and used in accordance with the Hiroshima University guidelines for the use and care of experimental animals.

### Microscopy

For human cell experiments, cells were moved to collagen-coated glass-bottom 24-well plates and fixed with 4% paraformaldehyde in PBS. Fluorescence was observed and cell images were captured with a 488-nm laser using a confocal laser-scanning microscope (Olympus FV-1000D). For *B. mori* experiments, fluorescence was observed using a fluorescence stereomicroscope (Olympus SZX16). For *X. laevis* experiments, fluorescence was observed using a fluorescence stereomicroscope (Leica MZ10F).

### Genomic PCR and DNA sequencing

A DNeasy Blood and Tissue kit (Qiagen) was used to extract genomic DNA from cell pellets, frog embryos and silkworm larvae or adults. Genomic PCR was performed using KOD FX (Toyobo), KOD FX Neo (Toyobo) or LA *Taq* (Takara) with the primers listed in Supplementary Table 6. For the human cell and silkworm experiments, the PCR products were subjected to direct DNA sequencing. For the *X. laevis* experiments, the PCR products were cloned and transformed into bacteria using a TOPO TA Cloning Kit with PCR2.1 TOPO (Life Technologies). Subsequently, colony PCR products were used as templates for DNA sequencing. DNA sequencing was performed using an ABI 3130xl Genetic analyzer (Life Technologies) with a BigDye Terminator v3.1 Cycle Sequencing Kit (Life Technologies).

### Off-target analyses

The PROGNOS tool (http://baolab.bme.gatech.edu/cgi-bin/prognos/prognos.cgi)^33^ was used to identify potential off-target sites for the *FBL* TALENs. Maximum mismatches per half-site and spacer lengths were set to 6 and 12–24, respectively. The CRISPR design tool (http://crispr.mit.edu/)^25^ was used to identify potential off-target sites for the three gRNAs against the genomic locus and CRIS-PITCh vector. Genomic regions around each candidate site were amplified by PCR using primers listed in Supplementary Table 7 and the sequence was confirmed by direct sequencing.

### Southern blot analyses

Southern blotting was carried out according to the previously report^34^ with some modifications, as described below. Five-μg aliquots of genomic DNA were digested with PstI, and 2 μg and 1 μg for the outer and the mNG probes, respectively, were resolved on 0.8% agarose gels. Digoxigenin-labelled DNA probes were made by PCR using KOD FX Neo (Toyobo) and DIG DNA labelling mix (Roche) with primers listed in Supplementary Table 6. Membrane transfer (Hybond-N+; GE Healthcare), ultraviolet cross-linking (120 mJ cm^−2^), pre-hybridization and hybridization were performed according to the instructions for DIG Easy Hyb Granules (Roche). The CDP-Star Detection Reagent (Roche) was used to develop the membrane, following the manufacturer’s instructions. The chemiluminescent signal was detected using Amersham Hyperfilm ECL (GE Healthcare).

## Author contributions

T.T., T.S. and K.T.S. designed the work. S.N., T.T., Y.S., S.K., T.S. and K.T.S. performed the experiments. T.T., T.S. and K.T.S. wrote the manuscript with support from all the authors. N.S., M.O., T.D. and H.S. provided instructions. T.Y. supervised the work.

## Additional information

**Accession codes**: Sequences of TAL-PITCh, CRIS-PITCh, and HR vectors have been deposited in the NCBI Genbank nucleotide database under accession codes LC008486, LC008487, LC008488, LC008489, LC008490, LC008491 and LC008492.

**How to cite this article**: Nakade, S. *et al.* Microhomology-mediated end-joining-dependent integration of donor DNA in cells and animals using TALENs and CRISPR/Cas9. *Nat. Commun.* 5:5560 doi: 10.1038/ncomms6560 (2014).

## Supplementary Material

Supplementary Information Supplementary Figures 1-12, Supplementary Tables 1-7.
