# Supplementary material for: Microhomology-mediated end-joining-dependent integration of donor DNA in cells and animals using TALENs and CRISPR/Cas9
Source: Nat Commun. 2014 Nov 20;5:5560. doi: 10.1038/ncomms6560 (PMC4263139; doi:10.1038/ncomms6560)
Supplement: Supplementary Information — Supplementary Figures 1-12, Supplementary Tables 1-7. [file ncomms6560-s1.pdf]

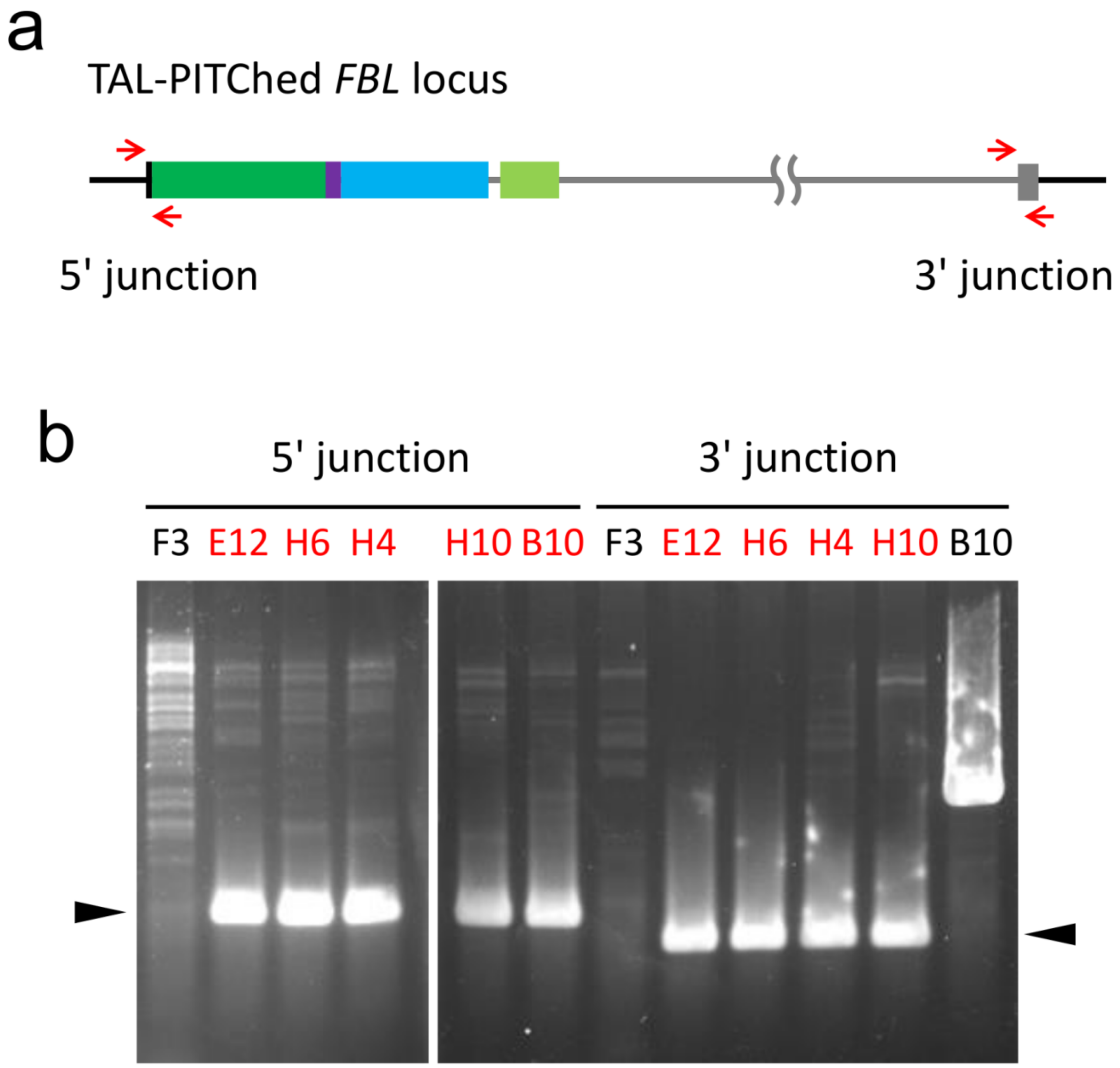

Supplementary Figure 1 Genomic PCR analysis of TAL-PITChed cell clones at the *FBL* locus. (**a**) Schematic illustration of the amplified genomic regions. Red arrows indicate primer annealing sites. (**b**) Gel images of the PCR products. Arrowheads indicate the expected positions of the amplicons from knocked-in alleles. Red letters indicate clone IDs showing the correct knockin bands.

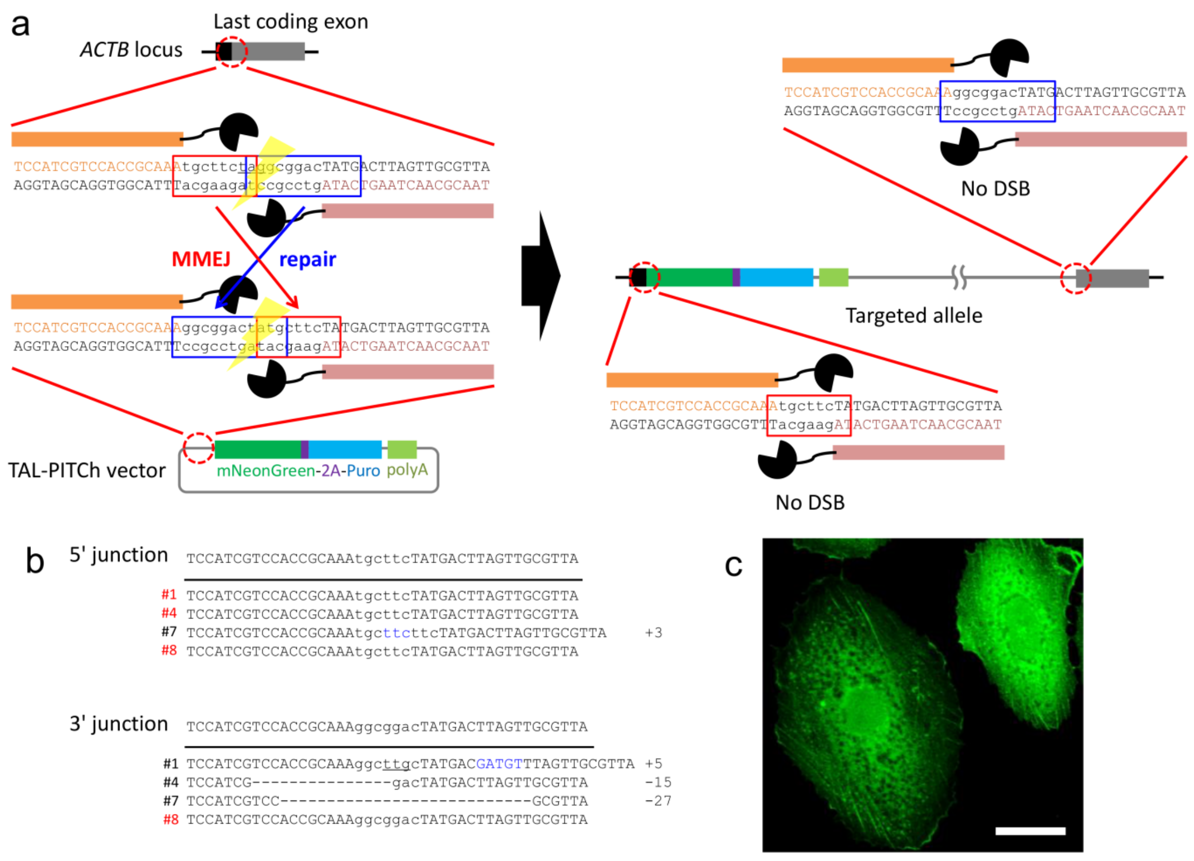

Supplementary Figure 2 TAL-PITCh at the *ACTB* locus in HeLa cells. (**a**) Schematic illustration of TAL-PITCh at the *ACTB* locus. Orange and pink letters indicate the left and right TALEN target sites, respectively. Red and blue boxes indicate the microhomologous sequences. The stop codon is underlined. (**b**) Sequences of knocked-in clones. The intended knocked-in sequence is shown at the top. TALEN target sites are shown in capital letters. Dashes indicate deletions. Blue letters indicate insertions. Substitutions are underlined. Red letters indicate correctly knocked-in clones. (**c**) Confocal laser scanning microscopy image of knocked-in cells (clone #8). Scale bar: 30 μm.

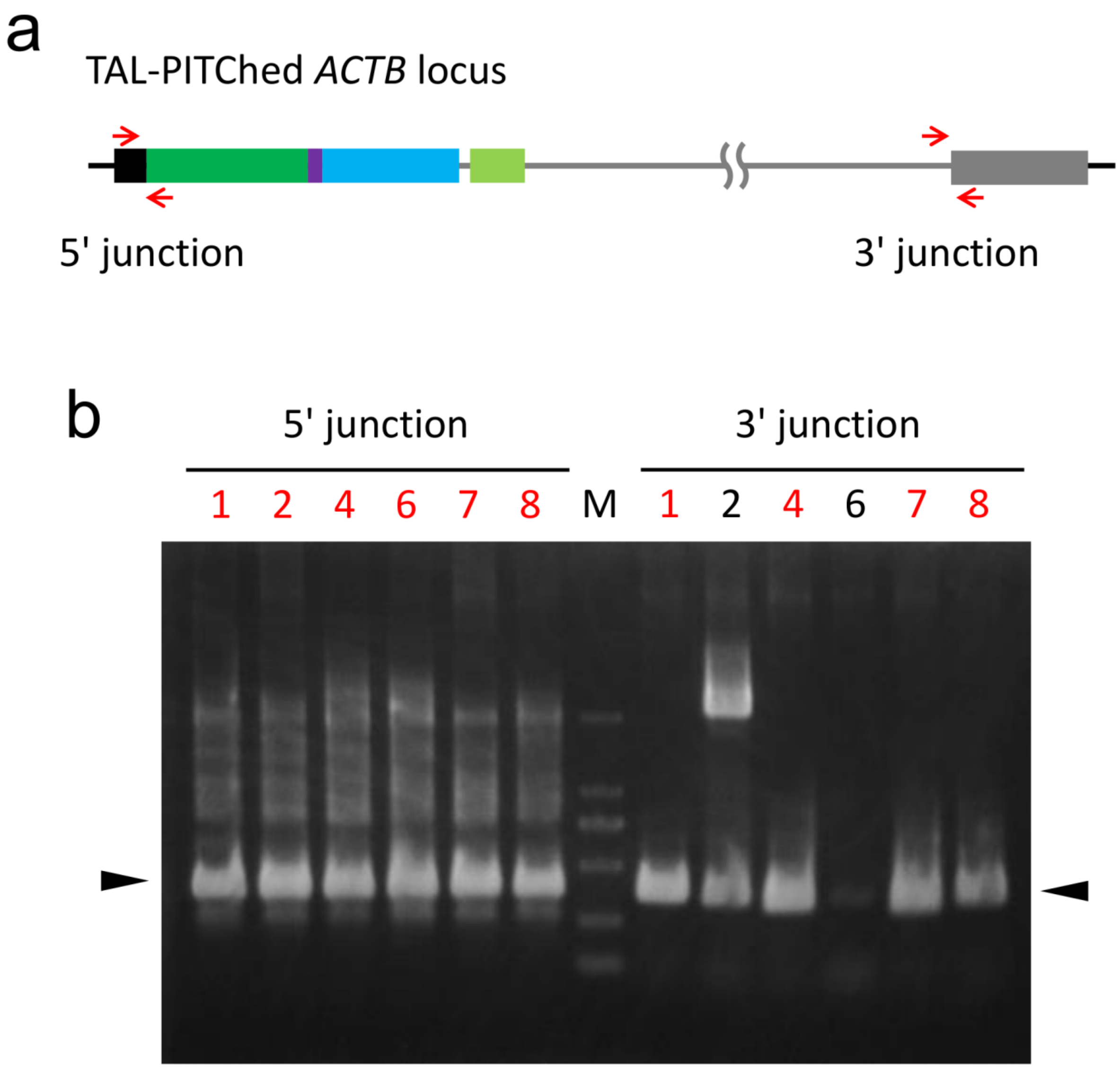

Supplementary Figure 3 Genomic PCR analysis of TAL-PITChed cell clones at the *ACTB* locus. (**a**) Schematic illustration of the amplified genomic regions. Red arrows indicate primer annealing sites. (**b**) A gel image of the PCR products. Arrowheads indicate the expected positions of the amplicons from knocked-in alleles. Red letters indicate clone IDs showing the correct knockin bands. M, Wide-Range DNA Ladder (100–2,000 bp) (Takara).

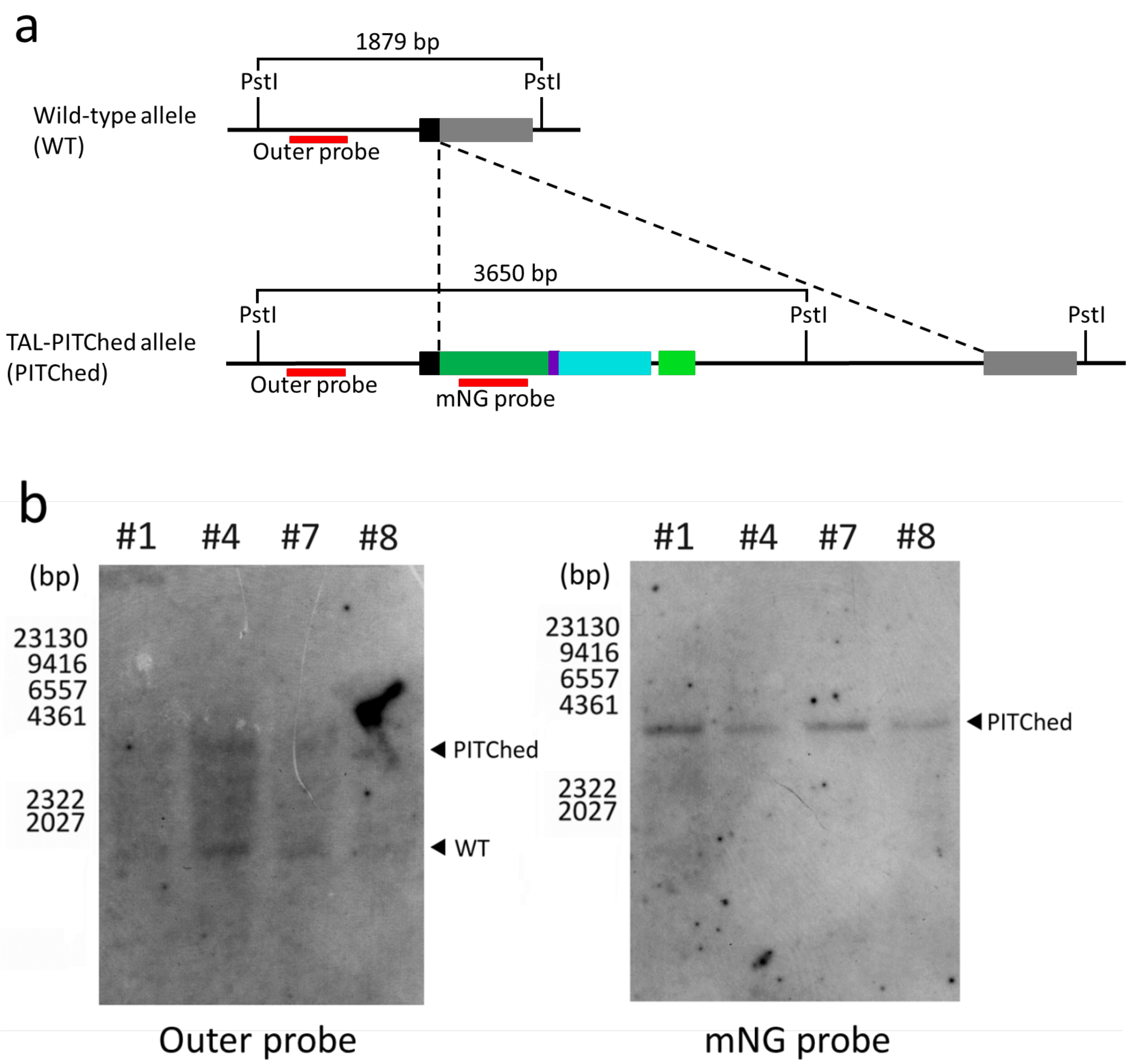

Supplementary Figure 4 Southern blot analysis for TAL-PITChed cell clones at the *ACTB* locus. (**a**) Schematic illustration of wild-type and TAL-PITChed *ACTB* locus. Two probe sites for endogenous (Outer probe) and exogenous (mNG probe) sequences are shown as red bars. mNG, mNeonGreen. (**b**) Chemiluminescent images of Southern blotting experiments. Clone IDs shown on the top of each panel are identical to Supplementary Figs. 2 and 3.

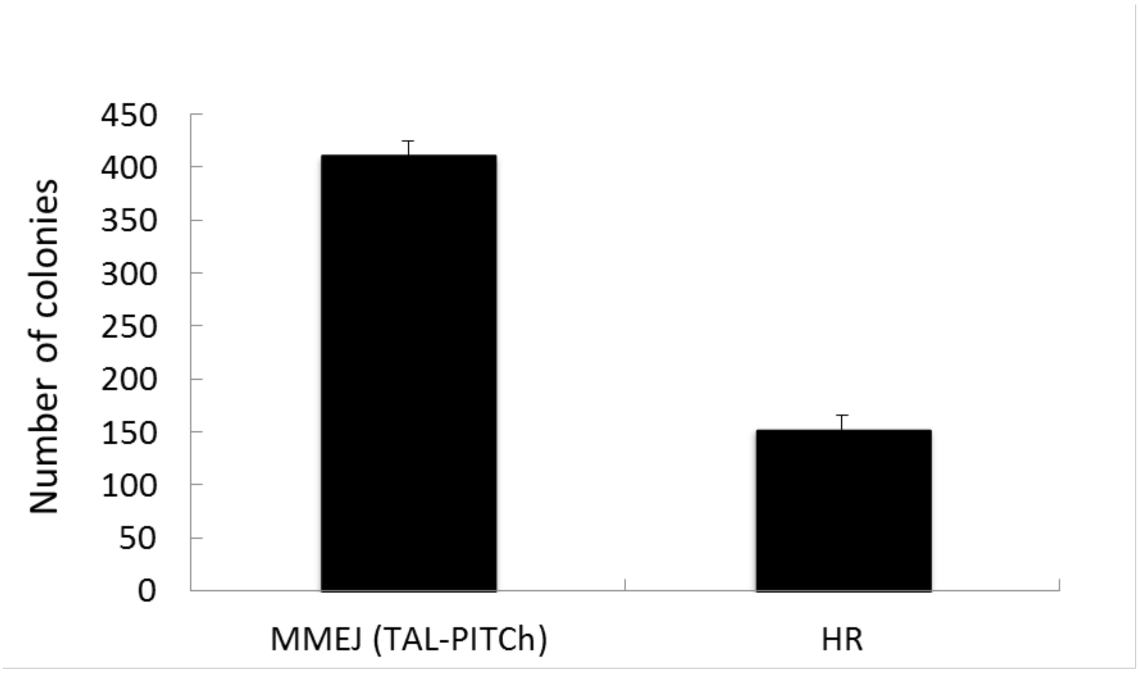

Supplementary Figure 5 Comparison of colony-forming efficiencies between MMEJ- and HR-mediated gene knockin at the *ACTB* locus in HeLa cells. For the MMEJ- or HR-mediated knockin, TALENs and TAL-PITCh vector or HR vector containing 1-kb homology arms were cotransfected and selected by puromycin. Numbers of all the colonies formed in 100-mm dishes showing fluorescence were counted. Data are expressed as means ±SEM (n=3).

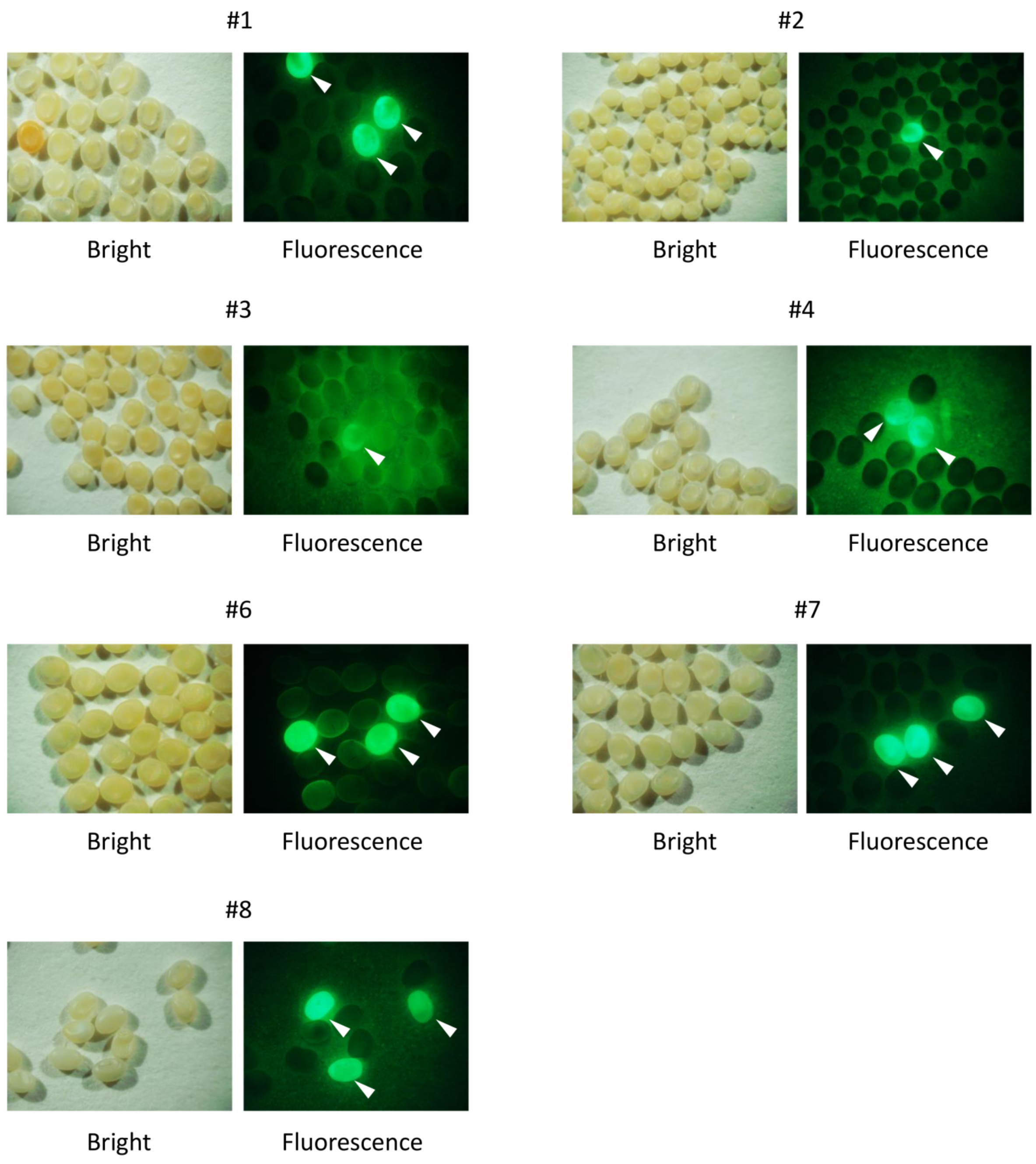

Supplementary Figure 6 EGFP expression in the G_1 embryos of TAL-PITChed silkworms. Batches with EGFP-positive embryos, excluding #5, are shown. White arrowheads indicate EGFP-positive embryos.

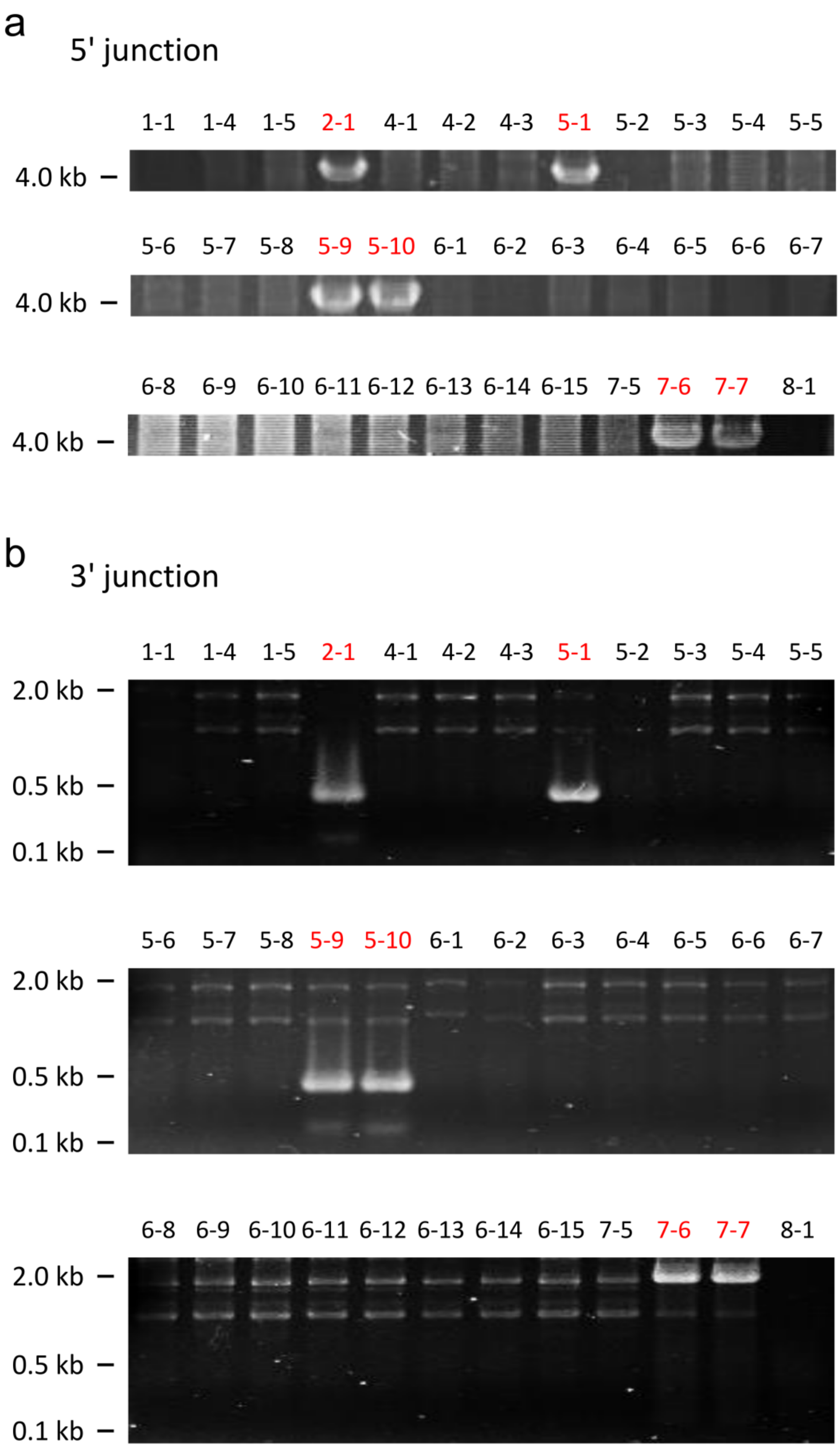

Supplementary Figure 7 Genomic PCR analysis of TAL-PITChed worms. Gel images of the PCR products at the 5′ (**a**) and 3′ (**b**) junctions. Red letters indicate worm IDs showing the knockin bands.

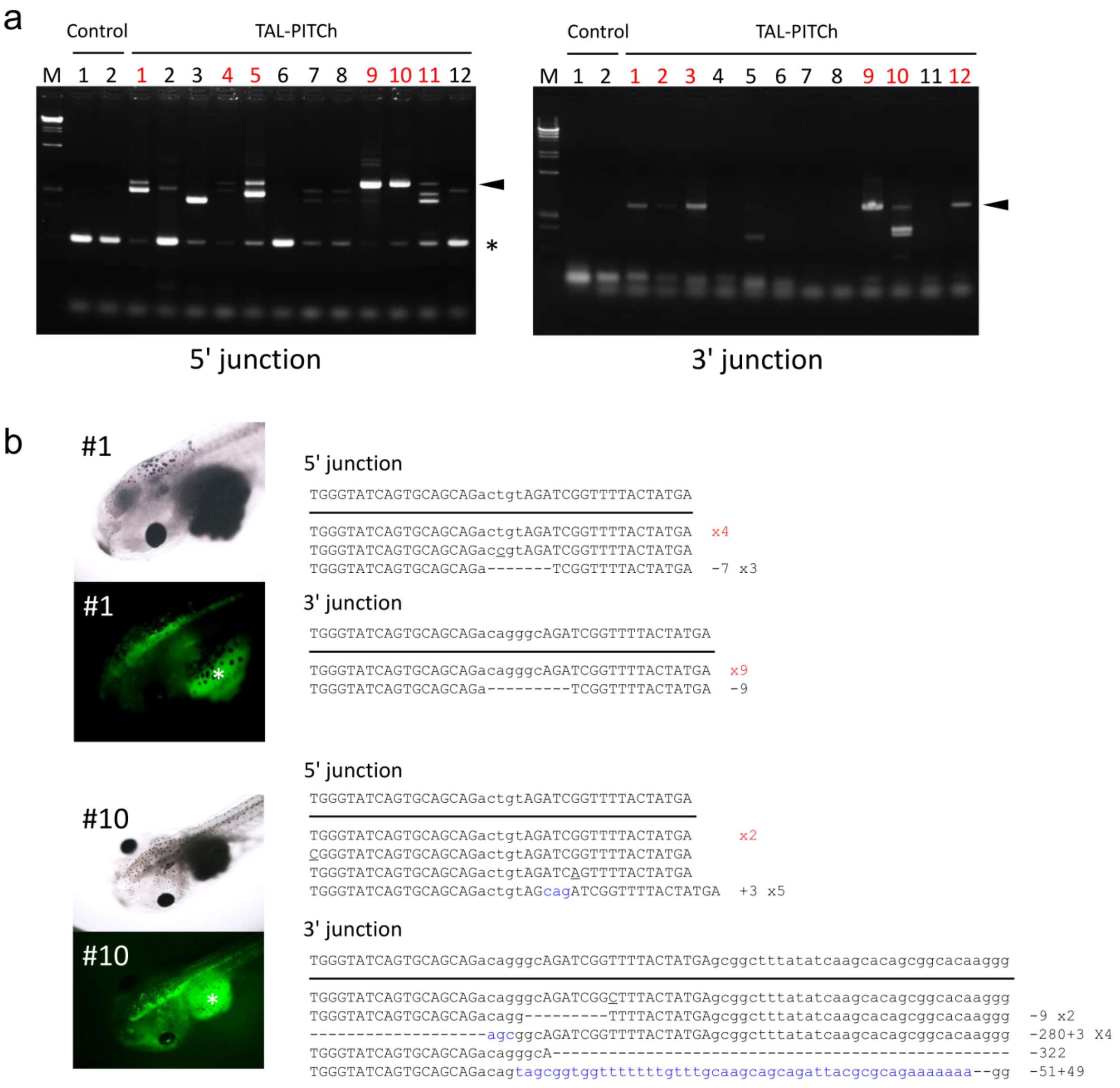

Supplementary Figure 8 Genomic PCR and sequencing analysis of TAL-PITChed embryos at the *no29* locus. (**a**) Gel images of the PCR products. Arrowheads indicate the expected positions of the amplicons from knocked-in alleles. Asterisks indicate non-specific bands. Red letters indicate clone IDs showing the correct knockin bands. M, λDNA/HindIII+pUC/HinfI marker. (**b**) Microscopy images and sequences of the embryos #1 and #10. Asterisks indicate yolk autofluorescence. Red letters indicate correctly knocked-in alleles. Blue letters indicate insertions. Dashes indicate deletions. Substitutions are underlined.

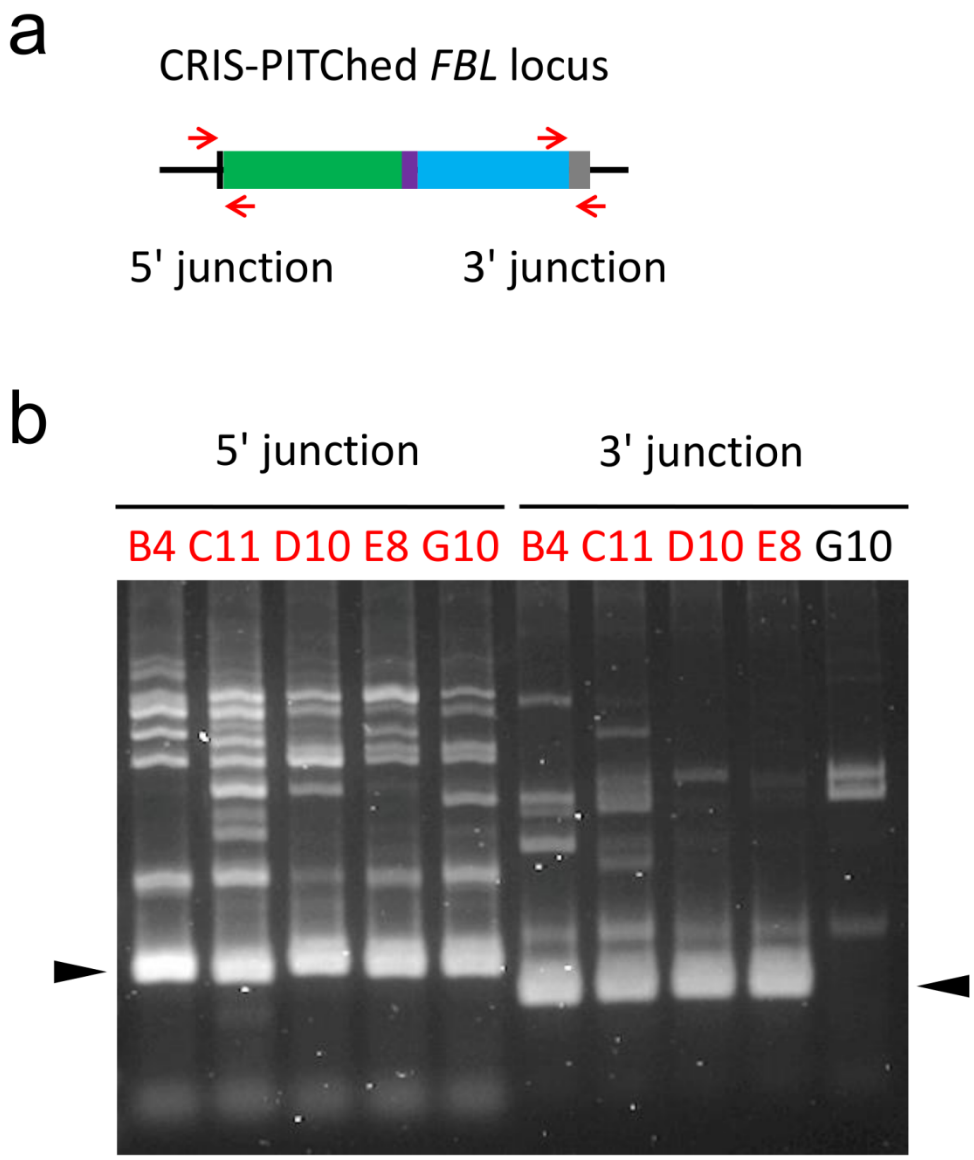

Supplementary Figure 9 Genomic PCR analysis of CRIS-PITChed cell clones at the *FBL* locus. (**a**) Schematic illustration of the amplified genomic regions. Red arrows indicate primer annealing sites. (**b**) A gel image of the PCR products. Arrowheads indicate the expected positions of the amplicons from knocked-in alleles. Red letters indicate clone IDs showing the correct knockin bands.

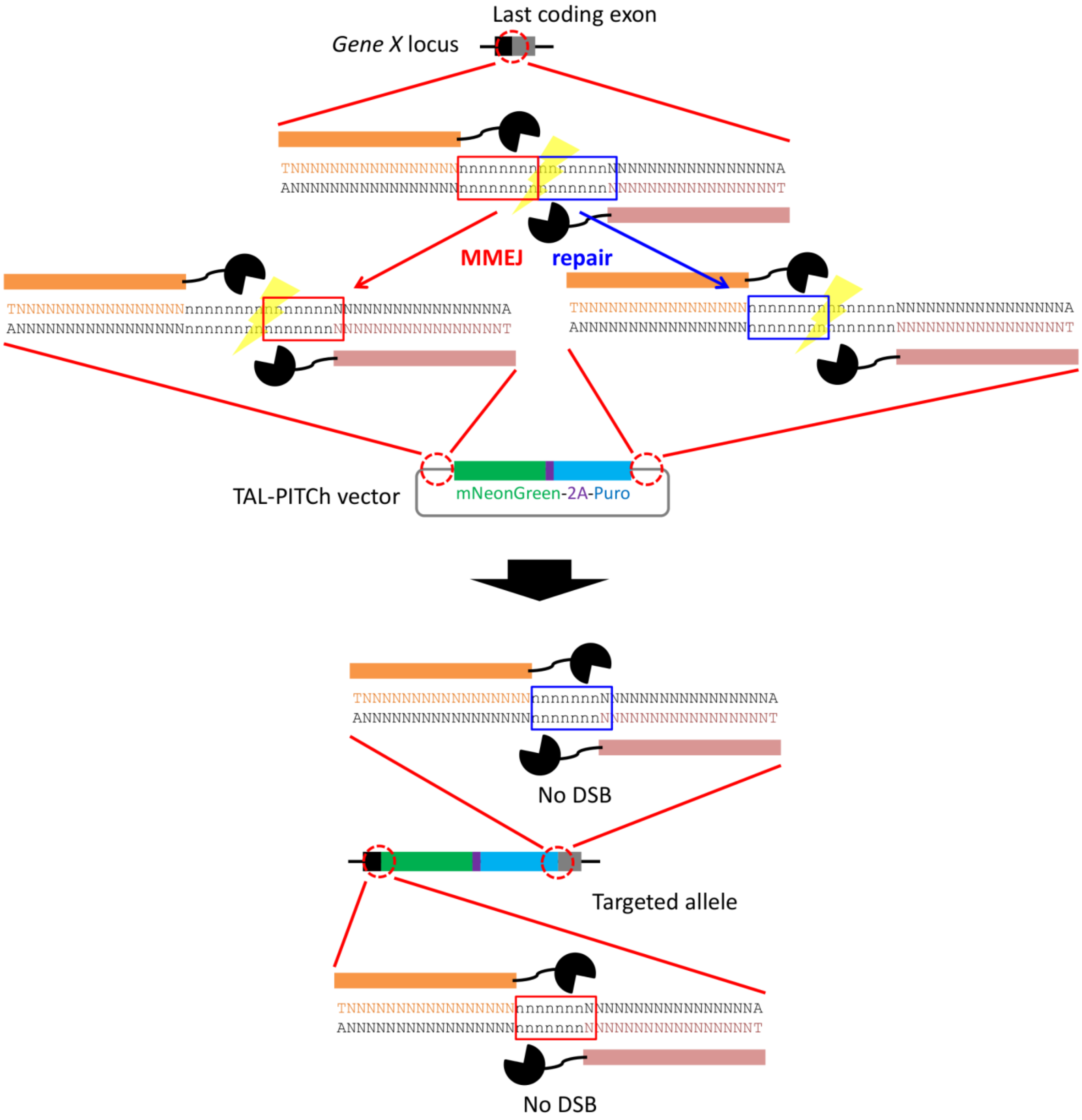

Supplementary Figure 10 Possible design of TAL-PITCh for cassette integration. For TAL-PITCh-mediated cassette integration, two TALEN target sites should be added at both ends of the cassette in the TAL-PITCh vector. The same TALEN set can be used to target three loci on the genome and on the TAL-PITCh vector. Orange and pink letters indicate the left and right TALEN target sites, respectively. Red and blue boxes indicate the microhomologous sequences.

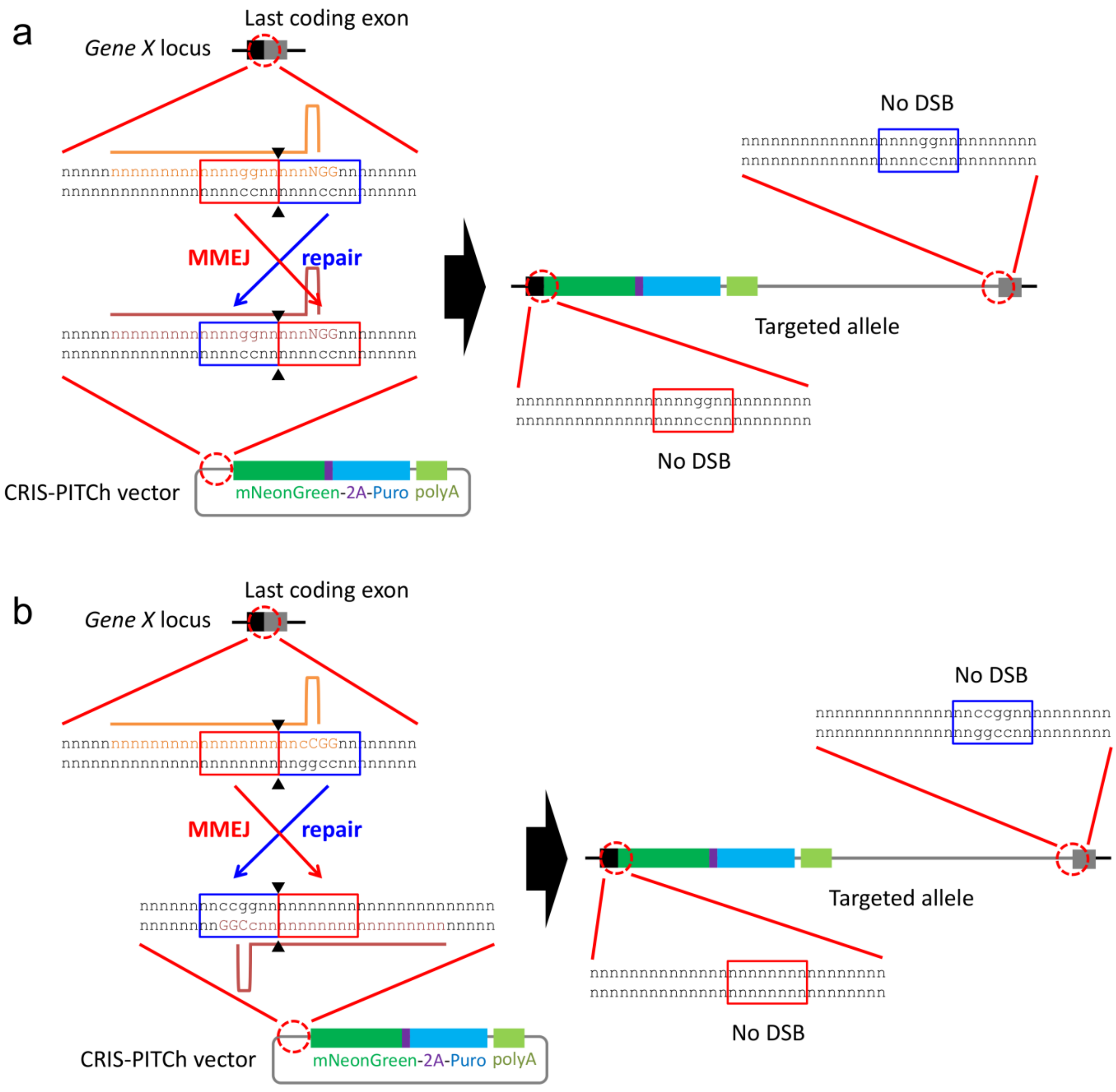

Supplementary Figure 11 Possible design of CRIS-PITCh for full plasmid integration. For CRIS-PITCh-mediated full plasmid integration, a single CRISPR/Cas9 site targeting the sense (**a**) or antisense (**b**) strand should be added to the CRIS-PITCh vector. The limitation of the genomic target sequence depends on which strand is targeted. Orange and pink letters indicate the gRNA target sites. Red and blue boxes indicate the microhomologous sequences.

>TAL-PITCh vector for the *FBL* gene

CTCATGACCAAAATCCCTTAACGTGAGTTACGCGCGCGTCGTTCCACTGAGCGTCAGACCCCGTAGAAAAGATCAAAGGATCTTCTTGAGATCCTTTTTT
TCTGCGCGTAATCTGCTGCTTGCAAACAAAAAAACCACCGCTACCAGCGGTGGTTTGTTTGCCGGATCAAGAGCTACCAACTCTTTTTCCGAAGGTAACT
GGCTTCAGCAGAGCGCAGATACCAAATACTGTTCTTCTAGTGTAGCCGTAGTTAGCCCACCACTTCAAGAACTCTGTAGCACCGCCTACATACCTCGCTC
TGCTAATCCTGTTACCAGTGGCTGCTGCCAGTGGCGATAAGTCGTGTCTTACCGGGTTGGACTCAAGACGATAGTTACCGGATAAGGCGCAGCGGTCGGG
CTGAACGGGGGGTTCGTGCACACAGCCCAGCTTGGAGCGAACGACCTACACCGAACTGAGATACCTACAGCGTGAGCTATGAGAAAGCGCCACGCTTCCC
GAAGGGAGAAAGGCGGACAGGTATCCGGTAAGCGGCAGGGTCGGAACAGGAGAGCGCACGAGGGAGCTTCCAGGGGGAAACGCCTGGTATCTTTATAGTC
CTGTCGGGTTTCGCCACCTCTGACTTGAGCGTCGATTTTTGTGATGCTCGTCAGGGGGGCGGAGCCTATGGAAAAACGCCAGCAACGCGGCCTTTTTACG
GTTCCTGGCCTTTTGCTGGCCTTTTGCTCACATGTTCTTTCCTGCGTTATCCCCTGATTCTGTGGATAACCGTATTACCGCCTTTGAGTGAGCTGATACC
GCTCGCCGCAGCCGAACGACCGAGCGCAGCGAGTCAGTGAGCGAGGAAGCGGAAGGCGAGAGTAGGGAACTGCCAGGCATCAAACTAAGCAGAAGGCCCC
TGACGGATGGCCTTTTTGCGTTTCTACAAACTCTTTCTGTGTTGTAAAACGACGGCCAGTCTTAAGCTCGGGCCCCCTGGGCGGTTCTGATAACGAGTAA
TCGTTAATCCGCAAATAACGTAAAAACCCGCTTCGGCGGGTTTTTTTATGGGGGGAGTTTAGGGAAAGAGCATTTGTCAGAATATTTAAGGGCGCCTGTC
ACTTTGCTTGATATATGAGAATTATTTAACCTTATAAATGAGAAAAAAGCAACGCACTTTAAATAAGATACGTTGCTTTTTCGATTGATGAACACCTATA
ATTAAACTATTCATCTATTATTTATGATTTTTTGTATATACAATATTTCTAGTTTGTTAAAGAGAATTAAGAAAATAAATCTCGAAAATAATAAAGGGAA
AATCAGTTTTTGATATCAAAATTATACATGTCAACGATAATACAAAATATAATACAAACTATAAGATGTTATCAGTATTTATTATCATTTAGAATAAATT
TTGTGTCGCCCTTAATTGTGAGCGGATAACAATTACGAGCTTCATGCACAGTGGCGTTGACATTGATTATTGACTAGTTATTAATAGTAATCAATTACGG
GGTCATTAGTTCATAGCCCATATATGGAGTTCCGCGTTACTCTCACAGGCCACCCCCAACTGAAGAAGGTGAAGTTCAGCGCTGTCAGGAGGGGATCCA
TGGTGAGTAAGGGAGAGGAAGATAATATGGCCTCCCTTCCCGCTACGCACGAACTCCACATCTTCGGGTCAATCAACGGTGTTGACTTCGACATGGTGGG
CCAGGGCACCGGCAATCCCAATGACGGATACGAAGAACTCAATTTGAAGAGTACAAAGGGCGATCTCCAATTCTCACCTTGGATTCTGGTTCCCCACATT
GGATACGGATTTCATCAGTACCTGCCGTACCCCGATGGGATGAGCCCATTTCAGGCTGCAATGGTAGATGGTAGCGGTTACCAAGTACACCGAACTATGC
AATTTGAGGACGGTGCCTCACTGACAGTGAACTATCGGTATACTTACGAAGGAAGCCACATCAAGGGAGAGGCACAGGTCAAAGGAACCGGATTTCCAGC
CGACGGGCCAGTCATGACAAACTCCCTGACCGCCGCAGATTGGTGCCGCAGCAAAAAGACCTATCCAAATGACAAGACCATTATCTCGACATTCAAATGG
AGCTACACCACCGGAAACGGCAAACGCTATCGGTCTACCGCCAGGACAACCTACACATTTGCAAAACCTATGGCCGCAAACTATCTGAAAAACCAGCCGA
TGTATGTGTTCCGAAAGACGGAATTAAAACACTCGAAAACAGAACTAAACTTTAAAGAGTGGCAGAAAGCCTTTACCGACGTAATGGGCATGGACGAGCT
GTATAAGGGAAGCGGAGAGGGCAGAGGAAGTCTGCTAACATGCGGTGACGTCGAGGAGAATCCTGGACCTATGACCGAGTACAAGCCCACGGTGCGCCTC
GCCACCCGCGACGACGTCCCCCGGGCCGTACGCACCCTCGCCGCCGCGTTCGCCGACTACCCCGCCACGCGCCACACCGTCGACCCGGACCGCCACATCG
AGCGGGTCACCGAGCTGCAAGAACTCTTCCTCACGCGCGTCGGGCTCGACATCGGCAAGGTGTGGGTCGCGGACGACGGCGCCGCGGTGGCGGTCTGGAC
CACGCCGGAGAGCGTCGAAGCGGGGGCGGTGTTCGCCGAGATCGGCCCGCGCATGGCCGAGTTGAGCGGTTCCCGGCTGGCCGCGCAGCAACAGATGGAA
GGCCTCCTGGCGCCGCACCGGCCCAAGGAGCCCGCGTGGTTCCTGGCCACCGTCGGCGTCTCGCCCGACCACCAGGGCAAGGGTCTGGGCAGCGCCGTCG
TGCTCCCCGGAGTGGAGGCGGCCGAGCGCGCCGGGGTGCCCGCCTTCCTGGAGACCTCCGCGCCCCGCAACCTCCCCTTCTACGAGCGGCTCGGCTTCAC
CGTCACCGCCGACGTCGAGGTGCCCGAAGGACCGCGCACCTGGTGCATGACCCGCAAGCCCGGTGCCTGATCTAGAATGCTGATGGGCTAGCAAAATCAG
CCTCGACTGTGCCTTCTAGTTGCCAGCCATCTGTTGTTTGCCCCTCCCCCGTGCCTTCCTTGACCCTGGAAGGTGCCACTCCCACTGTCCTTTCCTAATA
AAATGAGGAAATTGCATCACAACACTCAACCCTATCTCGGTCTATTCTTTTGATTTATAAGGGATTTTGCCGATTTCGGCCTATTGGTTAAAAAATGAGC
TGATTTAACAAAAATTTAACGCGAATTAATTCTGTGGAATGTGTGTCAGTTAGGGTGTGGAAAGTCCCCAGGCTCCCCAGCAGGCAGAAGTATGCAAAGC
ATGCATCTCAATTAGTCAGCAACCAGGTGTGGAAAGTCCCCAGGCTCCCCAGCAGGCAGAAGTATGCAAAGCATGCATCTCAATTAGTCAGCAACCATAG
TCCCGCCCCTAACTCCGCCCATCCCGCCCCTAACTCCGCCCAGTTCCGCCCATTCTCCGCCCCATGGCTGACTAATTTTTTTTATTTATGCAGAGGCCGA
GGCCGCCTCTGCCTCTGAGCTATTCCAGAAGTAGTGAGGAGGCTTTTTTGGAGGCCTAGGCTTTTGCAAAAAGCTCCCGGGAGCTTGTATATCCATTTTC
GGATCTGATCAGCACGTGATGAAAAAGCCTGAACTCACCGCGACGTCTGTCGAGAAGTTTCTGATCGAAAAGTTCGACAGCGTTTCCGACCTGATGCAGC
TCTCGGAGGGCGAAGAATCTCGTGCTTTCAGCTTCGATGTAGGAGGGCGTGGATATGTCCTGCGGGTAAATAGCTGCGCCGATGGTTTCTACAAAGATCG
TTATGTTTATCGGCACTTTGCATCGGCCGCGCTCCCGATTCCGGAAGTGCTTGACATTGGGGAATTCAGCGAGAGCCTGACCTATTGCATCTCCCGCCGT
GCACAGGGTGTCACGTTGCAAGACCTGCCTGAAACCGAACTGCCCGCTGTTCTGCAGCCGGTCGCGGAGGCCATGGATGCGATCGCTGCGGCCGATCTTA
GCCAGACGAGCGGGTTCGGCCCATTCGGACCGCAAGGAATCGGTCAATACACTACATGGCGTGATTTCATATGCGCGATTGCTGATCCCCATGTGTATCA
CTGGCAAACTGTGATGGACGACACCGTCAGTGCGTCCGTCGCGCAGGCTCTCGATGAGCTGATGCTTTGGGCCGAGGACTGCCCCGAAGTCCGGCACCTC
GTGCACGCGGATTTCGGCTCCAACAATGTCCTGACGGACAATGGCCGCATAACAGCGGTCATTGACTGGAGCGAGGCGATGTTCGGGGATTCCCAATACG
AGGTCGCCAACATCTTCTTCTGGAGGCCGTGGTTGGCTTGTATGGAGCAGCAGACGCGCTACTTCGAGCGGAGGCATCCGGAGCTTGCAGGATCGCCGCG
GCTCCGGGCGTATATGCTCCGCATTGGTCTTGACCAACTCTATCAGAGCTTGGTTGACGGCAATTTCGATGATGCAGCTTGGGCGCAGGGTCGATGCGAC
GCAATCGTCCGATCCGGAGCCGGGACTGTCGGGCGTACACAAATCGCCCGCAGAAGCGCGGCCGTCTGGACCGATGGCTGTGTAGAAGTACTCGCCGATA
GTGGAAACCGACGCCCCAGCACTCGTCCGAGGGCAAAGGAATAGCACGTGCTACGAGATTTCGATTCCACCGCCGCCTTCTATGAAAGGTTGGGCTTCGG
AATCGTTTTCCGGGACGCCGGCTGGATGATCCTCCAGCGCGGGGATCTCATGCTGGAGTTCTTCGCCCACCCCAACTTGTTTATTGCAGCTTATAATGGT
TACAAATAAAGCAATAGCATCACAAATTTCACAAATAAAGCATTTTTTTCACTGCATTCTAGTTGTGGTTTGTCCAAACTCATCAATGTATCTTATCATG
TCTGTATACCGTCGACCTCTAGCTAGAGCTTGGCGTAATCATGGTCATTACCAATGCTTAATCAGTGAGGCACCTATCTCAGCGATCTGTCTATTTCGTT
CATCCATAGTTGCCTGACTCCCCGTCGTGTAGATAACTACGATACGGGAGGGCTTACCATCTGGCCCCAGCGCTGCGATGATACCGCGAGAACCACGCTC
ACCGGCTCCGGATTTATCAGCAATAAACCAGCCAGCCGGAAGGGCCGAGCGCAGAAGTGGTCCTGCAACTTTATCCGCCTCCATCCAGTCTATTAATTGT
TGCCGGGAAGCTAGAGTAAGTAGTTCGCCAGTTAATAGTTTGCGCAACGTTGTTGCCATCGCTACAGGCATCGTGGTGTCACGCTCGTCGTTTGGTATGG
CTTCATTCAGCTCCGGTTCCCAACGATCAAGGCGAGTTACATGATCCCCCATGTTGTGCAAAAAAGCGGTTAGCTCCTTCGGTCCTCCGATCGTTGTCAG
AAGTAAGTTGGCCGCAGTGTTATCACTCATGGTTATGGCAGCACTGCATAATTCTCTTACTGTCATGCCATCCGTAAGATGCTTTTCTGTGACTGGTGAG
TACTCAACCAAGTCATTCTGAGAATAGTGTATGCGGCGACCGAGTTGCTCTTGCCCGGCGTCAATACGGGATAATACCGCGCCACATAGCAGAACTTTAA
AAGTGCTCATCATTGGAAAACGTTCTTCGGGGCGAAAACTCTCAAGGATCTTACCGCTGTTGAGATCCAGTTCGATGTAACCCACTCGTGCACCCAACTG
ATCTTCAGCATCTTTTACTTTCACCAGCGTTTCTGGGTGAGCAAAAACAGGAAGGCAAAATGCCGCAAAAAAGGGAATAAGGGCGACACGGAAATGTTGA
ATACTCATATTCTTCCTTTTTCAATATTATTGAAGCATTTATCAGGGTTATTGTCTCATGAGCGGATACATATTTGAATGTATTTAGAAAAATAAACAAA
TAGGGGTCAGTGTTACAACCAATTAACCAATTCTGAACATTATCGCGAGCCCATTTATACCTGAATATGGCTCATAACACCCCTTG

>TAL-PITCh vector for the *ACTB* gene

CTCATGACCAAAATCCCTTAACGTGAGTTACGCGCGCGTCGTTCCACTGAGCGTCAGACCCCGTAGAAAAGATCAAAGGATCTTCTTGAGATCCTTTTTT
TCTGCGCGTAATCTGCTGCTTGCAAACAAAAAAACCACCGCTACCAGCGGTGGTTTGTTTGCCGGATCAAGAGCTACCAACTCTTTTTCCGAAGGTAACT
GGCTTCAGCAGAGCGCAGATACCAAATACTGTTCTTCTAGTGTAGCCGTAGTTAGCCCACCACTTCAAGAACTCTGTAGCACCGCCTACATACCTCGCTC
TGCTAATCCTGTTACCAGTGGCTGCTGCCAGTGGCGATAAGTCGTGTCTTACCGGGTTGGACTCAAGACGATAGTTACCGGATAAGGCGCAGCGGTCGGG
CTGAACGGGGGGTTCGTGCACACAGCCCAGCTTGGAGCGAACGACCTACACCGAACTGAGATACCTACAGCGTGAGCTATGAGAAAGCGCCACGCTTCCC
GAAGGGAGAAAGGCGGACAGGTATCCGGTAAGCGGCAGGGTCGGAACAGGAGAGCGCACGAGGGAGCTTCCAGGGGGAAACGCCTGGTATCTTTATAGTC
CTGTCGGGTTTCGCCACCTCTGACTTGAGCGTCGATTTTTGTGATGCTCGTCAGGGGGGCGGAGCCTATGGAAAAACGCCAGCAACGCGGCCTTTTTACG
GTTCCTGGCCTTTTGCTGGCCTTTTGCTCACATGTTCTTTCCTGCGTTATCCCCTGATTCTGTGGATAACCGTATTACCGCCTTTGAGTGAGCTGATACC
GCTCGCCGCAGCCGAACGACCGAGCGCAGCGAGTCAGTGAGCGAGGAAGCGGAAGGCGAGAGTAGGGAACTGCCAGGCATCAAACTAAGCAGAAGGCCCC
TGACGGATGGCCTTTTTGCGTTTCTACAAACTCTTTCTGTGTTGTAAAACGACGGCCAGTCTTAAGCTCGGGCCCCCTGGGCGGTTCTGATAACGAGTAA
TCGTTAATCCGCAAATAACGTAAAAACCCGCTTCGGCGGGTTTTTTTATGGGGGGAGTTTAGGGAAAGAGCATTTGTCAGAATATTTAAGGGCGCCTGTC
ACTTTGCTTGATATATGAGAATTATTTAACCTTATAAATGAGAAAAAAGCAACGCACTTTAAATAAGATACGTTGCTTTTTCGATTGATGAACACCTATA
ATTAAACTATTCATCTATTATTTATGATTTTTTGTATATACAATATTTCTAGTTTGTTAAAGAGAATTAAGAAAATAAATCTCGAAAATAATAAAGGGAA
AATCAGTTTTTGATATCAAAATTATACATGTCAACGATAATACAAAATATAATACAAACTATAAGATGTTATCAGTATTTATTATCATTTAGAATAAATT
TTGTGTCGCCCTTAATTGTGAGCGGATAACAATTACGAGCTTCATGCACAGTGGCGTTGACATTGATTATTGACTAGTTATTAATAGTAATCAATTACGG
GGTCATTAGTTCATAGCCCATATATGGAGTTCCGCGTTACTCCATCGTCCACCGCAAAGGCGGACTATGCTTCTATGACTTAGTTGCGTTAGGATCCATG
GTGAGTAAGGGAGAGGAAGATAATATGGCCTCCCTTCCCGCTACGCACGAACTCCACATCTTCGGGTCAATCAACGGTGTTGACTTCGACATGGTGGGCC
AGGGCACCGGCAATCCCAATGACGGATACGAAGAACTCAATTTGAAGAGTACAAAGGGCGATCTCCAATTCTCACCTTGGATTCTGGTTCCCCACATTGG
ATACGGATTTCATCAGTACCTGCCGTACCCCGATGGGATGAGCCCATTTCAGGCTGCAATGGTAGATGGTAGCGGTTACCAAGTACACCGAACTATGCAA
TTTGAGGACGGTGCCTCACTGACAGTGAACTATCGGTATACTTACGAAGGAAGCCACATCAAGGGAGAGGCACAGGTCAAAGGAACCGGATTTCCAGCCG
ACGGGCCAGTCATGACAAACTCCCTGACCGCCGCAGATTGGTGCCGCAGCAAAAAGACCTATCCAAATGACAAGACCATTATCTCGACATTCAAATGGAG
CTACACCACCGGAAACGGCAAACGCTATCGGTCTACCGCCAGGACAACCTACACATTTGCAAAACCTATGGCCGCAAACTATCTGAAAAACCAGCCGATG
TATGTGTTCCGAAAGACGGAATTAAAACACTCGAAAACAGAACTAAACTTTAAAGAGTGGCAGAAAGCCTTTACCGACGTAATGGGCATGGACGAGCTGT
ATAAGGGAAGCGGAGAGGGCAGAGGAAGTCTGCTAACATGCGGTGACGTCGAGGAGAATCCTGGACCTATGACCGAGTACAAGCCCACGGTGCGCCTCGC

CACCCGCGACGACGTCCCCGGGCCGTACGCACCCTCGCCGCCGCGTTCGCCGACTACCCCGCCACGCGCCACACCGTCGACCCGGACCGCCACATCGAG
CGGGTCACCGAGCTGCAAGAACTCTTCCTCACGCGCGTCGGGCTCGACATCGGCAAGGTGTGGGTCGCGGACGACGGCGCCGCGGTGGCGGTCTGGACCA
CGCCGGAGAGCGTCGAAGCGGGGGCGGTGTTCGCCGAGATCGGCCCGCGCATGGCCGAGTTGAGCGGTTCCCGGCTGGCCGCGCAGCAACAGATGGAAGG
CCTCCTGGCGCCGCACCGGCCCAAGGAGCCCGCGTGGTTCCTGGCCACCGTCGGCGTCTCGCCCGACCACCAGGGCAAGGGTCTGGGCAGCGCCGTCGTG
CTCCCCGGAGTGGAGGCGGCCGAGCGCGCCGGGGTGCCCGCCTTCCTGGAGACCTCCGCGCCCCGCAACCTCCCCTTCTACGAGCGGCTCGGCTTCACCG
TCACCGCCGACGTCGAGGTGCCCGAAGGACCGCGCACCTGGTGCATGACCCGCAAGCCCGGTGCCTGATCTAGAATGCTGATGGGCTAGCAAAATCAGCC
TCGACTGTGCCTTCTAGTTGCCAGCCATCTGTTGTTTGCCCCTCCCCCGTGCCTTCCTTGACCCTGGAAGGTGCCACTCCCACTGTCCTTTCCTAATAAA
ATGAGGAAATTGCATCACAACACTCAACCCTATCTCGGTCTATTCTTTTGATTTATAAGGGATTTTGCCGATTTCGGCCTATTGGTTAAAAAATGAGCTG
ATTTAACAAAAATTTAACGCGAATTAATTCTGTGGAATGTGTGTCAGTTAGGGTGTGGAAAGTCCCCAGGCTCCCCAGCAGGCAGAAGTATGCAAAGCAT
GCATCTCAATTAGTCAGCAACCAGGTGTGGAAAGTCCCCAGGCTCCCCAGCAGGCAGAAGTATGCAAAGCATGCATCTCAATTAGTCAGCAACCATAGTC
CCGCCCCTAACTCCGCCCATCCCGCCCCTAACTCCGCCCAGTTCCGCCCATTCTCCGCCCCATGGCTGACTAATTTTTTTTATTTATGCAGAGGCCGAGG
CCGCCTCTGCCTCTGAGCTATTCCAGAAGTAGTGAGGAGGCTTTTTTGGAGGCCTAGGCTTTTGCAAAAAGCTCCCGGGAGCTTGTATATCCATTTTCGG
ATCTGATCAGCACGTGATGAAAAAGCCTGAACTCACCGCGACGTCTGTCGAGAAGTTTCTGATCGAAAAGTTCGACAGCGTTTCCGACCTGATGCAGCTC
TCGGAGGGCGAAGAATCTCGTGCTTTCAGCTTCGATGTAGGAGGGCGTGGATATGTCCTGCGGGTAAATAGCTGCGCCGATGGTTTCTACAAAGATCGTT
ATGTTTATCGGCACTTTGCATCGGCCGCGCTCCCGATTCCGGAAGTGCTTGACATTGGGGAATTCAGCGAGAGCCTGACCTATTGCATCTCCCGCCGTGC
ACAGGGTGTCACGTTGCAAGACCTGCCTGAAACCGAACTGCCCGCTGTTCTGCAGCCGGTCGCGGAGGCCATGGATGCGATCGCTGCGGCCGATCTTAGC
CAGACGAGCGGGTTCGGCCCATTCGGACCGCAAGGAATCGGTCAATACACTACATGGCGTGATTTCATATGCGCGATTGCTGATCCCCATGTGTATCACT
GGCAAACTGTGATGGACGACACCGTCAGTGCGTCCGTCGCGCAGGCTCTCGATGAGCTGATGCTTTGGGCCGAGGACTGCCCCGAAGTCCGGCACCTCGT
GCACGCGGATTTCGGCTCCAACAATGTCCTGACGGACAATGGCCGCATAACAGCGGTCATTGACTGGAGCGAGGCGATGTTCGGGGATTCCCAATACGAG
GTCGCCAACATCTTCTTCTGGAGGCCGTGGTTGGCTTGTATGGAGCAGCAGACGCGCTACTTCGAGCGGAGGCATCCGGAGCTTGCAGGATCGCCGCGGC
TCCGGGCGTATATGCTCCGCATTGGTCTTGACCAACTCTATCAGAGCTTGGTTGACGGCAATTTCGATGATGCAGCTTGGGCGCAGGGTCGATGCGACGC
AATCGTCCGATCCGGAGCCGGGACTGTCGGGCGTACACAAATCGCCCGCAGAAGCGCGGCCGTCTGGACCGATGGCTGTGTAGAAGTACTCGCCGATAGT
GGAAACCGACGCCCCAGCACTCGTCCGAGGGCAAAGGAATAGCACGTGCTACGAGATTTCGATTCCACCGCCGCCTTCTATGAAAGGTTGGGCTTCGGAA
TCGTTTTCCGGGACGCCGGCTGGATGATCCTCCAGCGCGGGGATCTCATGCTGGAGTTCTTCGCCCACCCCAACTTGTTTATTGCAGCTTATAATGGTTA
CAAATAAAGCAATAGCATCACAAATTTCACAAATAAAGCATTTTTTTCACTGCATTCTAGTTGTGGTTTGTCCAAACTCATCAATGTATCTTATCATGTC
TGTATACCGTCGACCTCTAGCTAGAGCTTGGCGTAATCATGGTCATTACCAATGCTTAATCAGTGAGGCACCTATCTCAGCGATCTGTCTATTTCGTTCA
TCCATAGTTGCCTGACTCCCCGTCGTGTAGATAACTACGATACGGGAGGGCTTACCATCTGGCCCCAGCGCTGCGATGATACCGCGAGAACCACGCTCAC
CGGCTCCGGATTTATCAGCAATAAACCAGCCAGCCGGAAGGGCCGAGCGCAGAAGTGGTCCTGCAACTTTATCCGCCTCCATCCAGTCTATTAATTGTTG
CCGGGAAGCTAGAGTAAGTAGTTCGCCAGTTAATAGTTTGCGCAACGTTGTTGCCATCGCTACAGGCATCGTGGTGTCACGCTCGTCGTTTGGTATGGCT
TCATTCAGCTCCGGTTCCCAACGATCAAGGCGAGTTACATGATCCCCCATGTTGTGCAAAAAAGCGGTTAGCTCCTTCGGTCCTCCGATCGTTGTCAGAA
GTAAGTTGGCCGCAGTGTTATCACTCATGGTTATGGCAGCACTGCATAATTCTCTTACTGTCATGCCATCCGTAAGATGCTTTTCTGTGACTGGTGAGTA
CTCAACCAAGTCATTCTGAGAATAGTGTATGCGGCGACCGAGTTGCTCTTGCCCGGCGTCAATACGGGATAATACCGCGCCACATAGCAGAACTTTAAAA
GTGCTCATCATTGGAAAACGTTCTTCGGGGCGAAAACTCTCAAGGATCTTACCGCTGTTGAGATCCAGTTCGATGTAACCCACTCGTGCACCCAACTGAT
CTTCAGCATCTTTTACTTTCACCAGCGTTTCTGGGTGAGCAAAAACAGGAAGGCAAAATGCCGCAAAAAAGGGAATAAGGGCGACACGGAAATGTTGAAT
ACTCATATTCTTCCTTTTTCAATATTATTGAAGCATTTATCAGGGTTATTGTCTCATGAGCGGATACATATTTGAATGTATTTAGAAAAATAAACAAATA
GGGGTCAGTGTTACAACCAATTAACCAATTCTGAACATTATCGCGAGCCCATTTATACCTGAATATGGCTCATAACACCCCTTG

>CRIS-PITCh vector for the *FBL* gene
CTCATGACCAAAATCCCTTAACGTGAGTTACGCGCGCGTCGTTCCACTGAGCGTCAGACCCCGTAGAAAAGATCAAAGGATCTTCTTGAGATCCTTTTTT
TCTGCGCGTAATCTGCTGCTTGCAAACAAAAAAACCACCGCTACCAGCGGTGGTTTGTTTGCCGGATCAAGAGCTACCAACTCTTTTTCCGAAGGTAACT
GGCTTCAGCAGAGCGCAGATACCAAATACTGTTCTTCTAGTGTAGCCGTAGTTAGCCCACCACTTCAAGAACTCTGTAGCACCGCCTACATACCTCGCTC
TGCTAATCCTGTTACCAGTGGCTGCTGCCAGTGGCGATAAGTCGTGTCTTACCGGGTTGGACTCAAGACGATAGTTACCGGATAAGGCGCAGCGGTCGGG
CTGAACGGGGGGTTCGTGCACACAGCCCAGCTTGGAGCGAACGACCTACACCGAACTGAGATACCTACAGCGTGAGCTATGAGAAAGCGCCACGCTTCCC
GAAGGGAGAAAGGCGGACAGGTATCCGGTAAGCGGCAGGGTCGGAACAGGAGAGCGCACGAGGGAGCTTCCAGGGGGAAACGCCTGGTATCTTTATAGTC
CTGTCGGGTTTCGCCACCTCTGACTTGAGCGTCGATTTTTGTGATGCTCGTCAGGGGGGCGGAGCCTATGGAAAAACGCCAGCAACGCGGCCTTTTTACG
GTTCCTGGCCTTTTGCTGGCCTTTTGCTCACATGTTCTTTCCTGCGTTATCCCCTGATTCTGTGGATAACCGTATTACCGCCTTTGAGTGAGCTGATACC
GCTCGCCGCAGCCGAACGACCGAGCGCAGCGAGTCAGTGAGCGAGGAAGCGGAAGGCGAGAGTAGGGAACTGCCAGGCATCAAACTAAGCAGAAGGCCCC
TGACGGATGGCCTTTTTGCGTTTCTACAAACTCTTTCTGTGTTGTAAAACGACGGCCAGTCTTAAGCTCGGGCCCCCTGGGCGGTTCTGATAACGAGTAA
TCGTTAATCCGCAAATAACGTAAAAACCCGCTTCGGCGGGTTTTTTTATGGGGGGAGTTTAGGGAAAGAGCATTTGTCAGAATATTTAAGGGCGCCTGTC
ACTTTGCTTGATATATGAGAATTATTTAACCTTATAAATGAGAAAAAAGCAACGCACTTTAAATAAGATACGTTGCTTTTTCGATTGATGAACACCTATA
ATTAAACTATTCATCTATTATTTATGATTTTTTGTATATACAATATTTCTAGTTTGTTAAAGAGAATTAAGAAAATAAATCTCGAAAATAATAAAGGGAA
AATCAGTTTTTGATATCAAAATTATACATGTCAACGATAATACAAAATATAATACAAACTATAAGATGTTATCAGTATTTATTATCATTTAGAATAAATT
TTGTGTCGCCCTTAATTGTGAGCGGATAACAATTACGAGCTTCATGCACAGTGGCGTTGACATTGATTATTGACTAGTTATTAATAGTAATCAATTACGG
GGTCATTAGTTCATAGCCCATATATGGAGTTCCGCGTTACATACCCGGGGCCACCCCACGGATCCATGGTGAGTAAGGGAGAGGAAGATAATATGGCCTC
CCTTCCCGCTACGCACGAACTCCACATCTTCGGGTCAATCAACGGTGTTGACTTCGACATGGTGGGCCAGGGCACCGGCAATCCCAATGACGGATACGAA
GAACTCAATTTGAAGAGTACAAAGGGCGATCTCCAATTCTCACCTTGGATTCTGGTTCCCCACATTGGATACGGATTTCATCAGTACCTGCCGTACCCCG
ATGGGATGAGCCCATTTCAGGCTGCAATGGTAGATGGTAGCGGTTACCAAGTACACCGAACTATGCAATTTGAGGACGGTGCCTCACTGACAGTGAACTA
TCGGTATACTTACGAAGGAAGCCACATCAAGGGAGAGGCACAGGTCAAAGGAACCGGATTTCCAGCCGACGGGCCAGTCATGACAAACTCCCTGACCGCC
GCAGATTGGTGCCGCAGCAAAAAGACCTATCCAAATGACAAGACCATTATCTCGACATTCAAATGGAGCTACACCACCGGAAACGGCAAACGCTATCGGT
CTACCGCCAGGACAACCTACACATTTGCAAAACCTATGGCCGCAAACTATCTGAAAAACCAGCCGATGTATGTGTTCCGAAAGACGGAATTAAAACACTC
GAAAACAGAACTAAACTTTAAAGAGTGGCAGAAAGCCTTTACCGACGTAATGGGCATGGACGAGCTGTATAAGGGAAGCGGAGAGGGCAGAGGAAGTCTG
CTAACATGCGGTGACGTCGAGGAGAATCCTGGACCTATGACCGAGTACAAGCCCACGGTGCGCCTCGCCACCCGCGACGACGTCCCCGGGCCGTACGCA
CCCTCGCCGCCGCGTTCGCCGACTACCCCGCCACGCGCCACACCGTCGACCCGGACCGCCACATCGAGCGGGTCACCGAGCTGCAAGAACTCTTCCTCAC
GCGCGTCGGGCTCGACATCGGCAAGGTGTGGGTCGCGGACGACGGCGCCGCGGTGGCGGTCTGGACCACGCCGGAGAGCGTCGAAGCGGGGGCGGTGTTC
GCCGAGATCGGCCCGCGCATGGCCGAGTTGAGCGGTTCCCGGCTGGCCGCGCAGCAACAGATGGAAGGCCTCCTGGCGCCGCACCGGCCCAAGGAGCCCG
CGTGGTTCCTGGCCACCGTCGGCGTCTCGCCCGACCACCAGGGCAAGGGTCTGGGCAGCGCCGTCGTGCTCCCCGGAGTGGAGGCGGCCGAGCGCGCCGG
GGTGCCCGCCTTCCTGGAGACCTCCGCGCCCCGCAACCTCCCCTTCTACGAGCGGCTCGGCTTCACCGTCACCGCCGACGTCGAGGTGCCCGAAGGACCG
CGCACCTGGTGCATGACCCGCAAGCCCGGTGCCTGACCAAGGTGCCCGGGTCTAGAATGCTGATGGGCTAGCAAAATCAGCCTCGACTGTGCCTTCTAGT
TGCCAGCCATCTGTTGTTTGCCCCTCCCCCGTGCCTTCCTTGACCCTGGAAGGTGCCACTCCCACTGTCCTTTCCTAATAAAATGAGGAAATTGCATCAC
AACACTCAACCCTATCTCGGTCTATTCTTTTGATTTATAAGGGATTTTGCCGATTTCGGCCTATTGGTTAAAAAATGAGCTGATTTAACAAAAATTTAAC
GCGAATTAATTCTGTGGAATGTGTGTCAGTTAGGGTGTGGAAAGTCCCCAGGCTCCCCAGCAGGCAGAAGTATGCAAAGCATGCATCTCAATTAGTCAGC
AACCAGGTGTGGAAAGTCCCCAGGCTCCCCAGCAGGCAGAAGTATGCAAAGCATGCATCTCAATTAGTCAGCAACCATAGTCCCGCCCCTAACTCCGCCC
ATCCCGCCCCTAACTCCGCCCAGTTCCGCCCATTCTCCGCCCCATGGCTGACTAATTTTTTTTATTTATGCAGAGGCCGAGGCCGCCTCTGCCTCTGAGC
TATTCCAGAAGTAGTGAGGAGGCTTTTTTGGAGGCCTAGGCTTTTGCAAAAAGCTCCCGGGAGCTTGTATATCCATTTTCGGATCTGATCAGCACGTGAT
GAAAAAGCCTGAACTCACCGCGACGTCTGTCGAGAAGTTTCTGATCGAAAAGTTCGACAGCGTTTCCGACCTGATGCAGCTCTCGGAGGGCGAAGAATCT
CGTGCTTTCAGCTTCGATGTAGGAGGGCGTGGATATGTCCTGCGGGTAAATAGCTGCGCCGATGGTTTCTACAAAGATCGTTATGTTTATCGGCACTTTG
CATCGGCCGCGCTCCCGATTCCGGAAGTGCTTGACATTGGGGAATTCAGCGAGAGCCTGACCTATTGCATCTCCCGCCGTGCACAGGGTGTCACGTTGCA
AGACCTGCCTGAAACCGAACTGCCCGCTGTTCTGCAGCCGGTCGCGGAGGCCATGGATGCGATCGCTGCGGCCGATCTTAGCCAGACGAGCGGGTTCGGC
CCATTCGGACCGCAAGGAATCGGTCAATACACTACATGGCGTGATTTCATATGCGCGATTGCTGATCCCCATGTGTATCACTGGCAAACTGTGATGGACG
ACACCGTCAGTGCGTCCGTCGCGCAGGCTCTCGATGAGCTGATGCTTTGGGCCGAGGACTGCCCCGAAGTCCGGCACCTCGTGCACGCGGATTTCGGCTC
CAACAATGTCCTGACGGACAATGGCCGCATAACAGCGGTCATTGACTGGAGCGAGGCGATGTTCGGGGATTCCCAATACGAGGTCGCCAACATCTTCTTC
TGGAGGCCGTGGTTGGCTTGTATGGAGCAGCAGACGCGCTACTTCGAGCGGAGGCATCCGGAGCTTGCAGGATCGCCGCGGCTCCGGGCGTATATGCTCC
GCATTGGTCTTGACCAACTCTATCAGAGCTTGGTTGACGGCAATTTCGATGATGCAGCTTGGGCGCAGGGTCGATGCGACGCAATCGTCCGATCCGGAGC
CGGGACTGTCGGGCGTACACAAATCGCCCGCAGAAGCGCGGCCGTCTGGACCGATGGCTGTGTAGAAGTACTCGCCGATAGTGGAAACCGACGCCCCAGC
ACTCGTCCGAGGGCAAAGGAATAGCACGTGCTACGAGATTTCGATTCCACCGCCGCCTTCTATGAAAGGTTGGGCTTCGGAATCGTTTTCCGGGACGCCG
GCTGGATGATCCTCCAGCGCGGGGATCTCATGCTGGAGTTCTTCGCCCACCCCAACTTGTTTATTGCAGCTTATAATGGTTACAAATAAAGCAATAGCAT

CACAAATTTCACAAATAAAGCATTTTTTTCACTGCATTCTAGTTGTGGTTTGTCCAAACTCATCAATGTATCTTATCATGTCTGTATACCGTCGACCTCT
AGCTAGAGCTTGGCGTAATCATGGTCATTACCAATGCTTAATCAGTGAGGCACCTATCTCAGCGATCTGTCTATTTCGTTCATCCATAGTTGCCTGACTC
CCCGTCGTGTAGATAACTACGATACGGGAGGGCTTACCATCTGGCCCCAGCGCTGCGATGATACCGCGAGAACCACGCTCACCGGCTCCGGATTTATCAG
CAATAAACCAGCCAGCCGGAAGGGCCGAGCGCAGAAGTGGTCCTGCAACTTTATCCGCCTCCATCCAGTCTATTAATTGTTGCCGGGAAGCTAGAGTAAG
TAGTTCGCCAGTTAATAGTTTGCGCAACGTTGTTGCCATCGCTACAGGCATCGTGGTGTCACGCTCGTCGTTTGGTATGGCTTCATTCAGCTCCGGTTCC
CAACGATCAAGGCGAGTTACATGATCCCCCATGTTGTGCAAAAAAGCGGTTAGCTCCTTCGGTCCTCCGATCGTTGTCAGAAGTAAGTTGGCCGCAGTGT
TATCACTCATGGTTATGGCAGCACTGCATAATTCTCTTACTGTCATGCCATCCGTAAGATGCTTTTCTGTGACTGGTGAGTACTCAACCAAGTCATTCTG
AGAATAGTGTATGCGGCGACCGAGTTGCTCTTGCCCGGCGTCAATACGGGATAATACCGCGCCACATAGCAGAACTTTAAAAGTGCTCATCATTGGAAAA
CGTTCTTCGGGGCGAAAACTCTCAAGGATCTTACCGCTGTTGAGATCCAGTTCGATGTAACCCACTCGTGCACCCAACTGATCTTCAGCATCTTTTACTT
TCACCAGCGTTTCTGGGTGAGCAAAAACAGGAAGGCAAAATGCCGCAAAAAAGGGAATAAGGGCGACACGGAAATGTTGAATACTCATATTCTTCCTTTT
TCAATATTATTGAAGCATTTATCAGGGTTATTGTCTCATGAGCGGATACATATTTGAATGTATTTAGAAAAATAAACAAATAGGGGTCAGTGTTACAACC
AATTAACCAATTCTGAACATTATCGCGAGCCCATTTATACCTGAATATGGCTCATAACACCCCTTG

>HR vector for the *ACTB* gene
CTCATGACCAAAATCCCTTAACGTGAGTTACGCGCGCGTCGTTCCACTGAGCGTCAGACCCCGTAGAAAAGATCAAAGGATCTTCTTGAGATCCTTTTTT
TCTGCGCGTAATCTGCTGCTTGCAAACAAAAAAACCACCGCTACCAGCGGTGGTTTGTTTGCCGGATCAAGAGCTACCAACTCTTTTTCCGAAGGTAACT
GGCTTCAGCAGAGCGCAGATACCAAATACTGTTCTTCTAGTGTAGCCGTAGTTAGCCCACCACTTCAAGAACTCTGTAGCACCGCCTACATACCTCGCTC
TGCTAATCCTGTTACCAGTGGCTGCTGCCAGTGGCGATAAGTCGTGTCTTACCGGGTTGGACTCAAGACGATAGTTACCGGATAAGGCGCAGCGGTCGGG
CTGAACGGGGGGTTCGTGCACACAGCCCAGCTTGGAGCGAACGACCTACACCGAACTGAGATACCTACAGCGTGAGCTATGAGAAAGCGCCACGCTTCCC
GAAGGGAGAAAGGCGGACAGGTATCCGGTAAGCGGCAGGGTCGGAACAGGAGAGCGCACGAGGGAGCTTCCAGGGGGAAACGCCTGGTATCTTTATAGTC
CTGTCGGGTTTCGCCACCTCTGACTTGAGCGTCGATTTTTGTGATGCTCGTCAGGGGGGCGGAGCCTATGGAAAAACGCCAGCAACGCGGCCTTTTTACG
GTTCCTGGCCTTTTGCTGGCCTTTTGCTCACATGTTCTTTCCTGCGTTATCCCCTGATTCTGTGGATAACCGTATTACCGCCTTTGAGTGAGCTGATACC
GCTCGCCGCAGCCGAACGACCGAGCGCAGCGAGTCAGTGAGCGAGGAAGCGGAAGGCGAGAGTAGGGAACTGCCAGGCATCAAACTAAGCAGAAGGCCCC
TGACGGATGGCCTTTTTGCGTTTCTACAAACTCTTTCTGTGTTGTAAAACGACGGCCAGTCTTAAGCTCGGGCCCCCTGGGCGGTTCTGATAACGAGTAA
TCGTTAATCCGCAAATAACGTAAAAACCCGCTTCGGCGGGTTTTTTTATGGGGGGAGTTTAGGGAAAGAGCATTTGTCAGAATATTTAAGGGCGCCTGTC
ACTTTGCTTGATATATGAGAATTATTTAACCTTATAAATGAGAAAAAAGCAACGCACTTTAAATAAGATACGTTGCTTTTTCGATTGATGAACACCTATA
ATTAAACTATTCATCTATTATTTATGATTTTTTGTATATACAATATTTCTAGTTTGTTAAAGAGAATTAAGAAAATAAATCTCGAAAATAATAAAGGGAA
AATCAGTTTTTGATATCAAAATTATACATGTCAACGATAATACAAAATATAATACAAACTATAAGATGTTATCAGTATTTATTATCATTTAGAATAAATT
TTGTGTCGCCCTTAATTGTGAGCGGATAACAATTACGAGCTTCATGCACAGTGGCGTTGACATTGATTATTGACTAGTTATTAATAGTAATCAATTACGG
GGTCATTAGTTCATAGCCCATATATGGAGTTCCGCGTTACATATGTCCCCAGTGGCTTCCCCAGTGTGACATGGTGTATCTCTGCCTTACAGATCATGTT
TGAGACCTTCAACACCCCAGCCATGTACGTTGCTATCCAGGCTGTGCTATCCCTGTACGCCTCTGGCCGTACCACTGGCATCGTGATGGACTCCGGTGAC
GGGGTCACCCACACTGTGCCCATCTACGAGGGGTATGCCCTCCCCCATGCCATCCTGCGTCTGGACCTGGCTGGCCGGGACCTGACTGACTACCTCATGA
AGATCCTCACCGAGCGCGGCTACAGCTTCACCACCACGGCCGAGCGGGAAATCGTGCGTGACATTAAGGAGAAGCTGTGCTACGTCGCCCTGGACTTCGA
GCAAGAGATGGCCACGGCTGCTTCCAGCTCCTCCCTGGAGAAGAGCTACGAGCTGCCTGACGGCCAGGTCATCACCATTGGCAATGAGCGGTTCCGCTGC
CCTGAGGCACTCTTCCAGCCTTCCTTCCTGGGTGAGTGGAGACTGTCTCCCGGCTCTGCCTGACATGAGGGTTACCCCTCGGGGCTGTGCTGTGGAAGCT
AAGTCCTGCCCTCATTTCCCTCTCAGGCATGGAGTCCTGTGGCATCCACGAAACTACCTTCAACTCCATCATGAAGTGTGACGTGGACATCCGCAAAGAC
CTGTACGCCAACACAGTGCTGTCTGGCGGCACCACCATGTACCCTGGCATTGCCGACAGGATGCAGAAGGAGATCACTGCCCTGGCACCCAGCACAATGA
AGATCAAGGTGGGTGTCTTTCCTGCCTGAGCTGACCTGGGCAGGTCGGCTGTGGGGTCCTGTGGTGTGTGGGGAGCTGTCACATCCAGGGTCCTCACTGC
CTGTCCCCTTCCCTCCTCAGATCATTGCTCCTCCTGAGCGCAAGTACTCCGTGTGGATCGGCGGCTCCATCCTGGCCTCGCTGTCCACCTTCCAGCAGAT
GTGGATCAGCAAGCAGGAGTATGACGAGTCCGGCCCCTCCATCGTCCACCGCAAATGCTTGTATGACTTAGTTGCGTTAGGATCCATGGTGAGTAAGGGA
GAGGAAGATAATATGGCCTCCCTTCCCGCTACGCACGAACTCCACATCTTCGGGTCAATCAACGGTGTTGACTTCGACATGGTGGGCCAGGGCACCGGCA
ATCCCAATGACGGATACGAAGAACTCAATTTGAAGAGTACAAAGGGCGATCTCCAATTCTCACCTTGGATTCTGGTTCCCCACATTGGATACGGATTTCA
TCAGTACCTGCCGTACCCCGATGGGATGAGCCCATTTCAGGCTGCAATGGTAGATGGTAGCGGTTACCAAGTACACCGAACTATGCAATTTGAGGACGGT
GCCTCACTGACAGTGAACTATCGGTATACTTACGAAGGAAGCCACATCAAGGGAGAGGCACAGGTCAAAGGAACCGGATTTCCAGCCGACGGGCCAGTCA
TGACAAACTCCCTGACCGCCGCAGATTGGTGCCGCAGCAAAAAGACCTATCCAAATGACAAGACCATTATCTCGACATTCAAATGGAGCTACACCACCGG
AAACGGCAAACGCTATCGGTCTACCGCCAGGACAACCTACACATTTGCAAAACCTATGGCCGCAAACTATCTGAAAAACCAGCCGATGTATGTGTTCCGA
AAGACGGAATTAAAACACTCGAAAACAGAACTAAACTTTAAAGAGTGGCAGAAAGCCTTTACCGACGTAATGGGCATGGACGAGCTGTATAAGGGAAGCG
GAGAGGGCAGAGGAAGTCTGCTAACATGCGGTGACGTCGAGGAGAATCCTGGACCTATGACCGAGTACAAGCCCACGGTGCGCCTCGCCACCCGCGACGA
CGTCCCCCGGGCCGTACGCACCCTCGCCGCCGCGTTCGCCGACTACCCCGCCACGCGCCACACCGTCGACCCGGACCGCCACATCGAGCGGGTCACCGAG
CTGCAAGAACTCTTCCTCACGCGCGTCGGGCTCGACATCGGCAAGGTGTGGGTCGCGGACGACGGCGCCGCGGTGGCGGTCTGGACCACGCCGGAGAGCG
TCGAAGCGGGGGCGGTGTTCGCCGAGATCGGCCCGCGCATGGCCGAGTTGAGCGGTTCCCGGCTGGCCGCGCAGCAACAGATGGAAGGCCTCCTGGCGCC
GCACCGGCCCAAGGAGCCCGCGTGGTTCCTGGCCACCGTCGGCGTCTCGCCCGACCACCAGGGCAAGGGTCTGGGCAGCGCCGTCGTGCTCCCCGGAGTG
GAGGCGGCCGAGCGCGCCGGGGTGCCCGCCTTCCTGGAGACCTCCGCGCCCCGCAACCTCCCCTTCTACGAGCGGCTCGGCTTCACCGTCACCGCCGACG
TCGAGGTGCCCGAAGGACCGCGCACCTGGTGCATGACCCGCAAGCCCGGTGCCTGAGCGGACTATGACTTAGTTGCGTTACACCCTTTCTTGACAAAACC
TAACTTGCGCAGAAAACAAGATGAGATTGGCATGGCTTTATTTGTTTTTTTTGTTTTGTTTTGGTTTTTTTTTTTTTTTTGGCTTGACTCAGGATTTAAA
AACTGGAACGGTGAAGGTGACAGCAGTCGGTTGGAGCGAGCATCCCCCAAAGTTCACAATGTGGCCGAGGACTTTGATTGCACATTGTTGTTTTTTTAAT
AGTCATTCCAAATATGAGATGCGTTGTTACAGGAAGTCCCTTGCCATCCTAAAAGCCACCCCACTTCTCTCTAAGGAGAATGGCCCAGTCCTCTCCCAAG
TCCACACAGGGGAGGTGATAGCATTGCTTTCGTGTAAATTATGTAATGCAAAATTTTTTTAATCTTCGCCTTAATACTTTTTTATTTTGTTTTATTTTGA
ATGATGAGCCTTCGTGCCCCCCCTTCCCCCTTTTTTGTCCCCCAACTTGAGATGTATGAAGGCTTTTGGTCTCCCTGGGAGTGGGTGGAGGCAGCCAGGG
CTTACCTGTACACTGACTTGAGACCAGTTGAATAAAAGTGCACACCTTAAAAATGAGGCCAAGTGTGACTTTGTGGTGTGGCTGGGTTGGGGGCAGCAGA
GGGTGAACCCTGCAGGAGGGTGAACCCTGCAAAAGGGTGGGGCAGTGGGGGCCAACTTGTCCTTACCCAGAGTGCAGGTGTGTGGAGATCCCTCCTGCCT
TGACATTGAGCAGCCTTAGAGGGTGGGGGAGGCTCAGGGGTCAGGTCTCTGTTCCTGCTTATTGGGGAGTTCCTGGCCTGGCCCTTCTATGTCTCCCCAG
GTACCCCAGTTTTTCTGGGTTCACCCAGAGTGCAGATGCTTGAGGAGGTGGGAAGGGACTATTTGGGGGTGTCTGGCTCAGGTGCCATGCCTCACTGGGG
CTGGTTGGCACCTGCATTTCCTGGGAGTGGGGCTGTCTCAGGGTAGCTGGGCACGGTGTTCCCTTTCTAGAATGCTGATGGGCTAGCAAAATCAGCCTCG
ACTGTGCCTTCTAGTTGCCAGCCATCTGTTGTTTGCCCCTCCCCCGTGCCTTCCTTGACCCTGGAAGGTGCCACTCCCACTGTCCTTTCCTAATAAAATG
AGGAAATTGCATCACAACACTCAACCCTATCTCGGTCTATTCTTTTGATTTATAAGGGATTTTGCCGATTTCGGCCTATTGGTTAAAAAATGAGCTGATT
TAACAAAAATTTAACGCGAATTAATTCTGTGGAATGTGTGTCAGTTAGGGTGTGGAAAGTCCCCAGGCTCCCCAGCAGGCAGAAGTATGCAAAGCATGCA
TCTCAATTAGTCAGCAACCAGGTGTGGAAAGTCCCCAGGCTCCCCAGCAGGCAGAAGTATGCAAAGCATGCATCTCAATTAGTCAGCAACCATAGTCCCG
CCCCTAACTCCGCCCATCCCGCCCCTAACTCCGCCCAGTTCCGCCCATTCTCCGCCCCATGGCTGACTAATTTTTTTTATTTATGCAGAGGCCGAGGCCG
CCTCTGCCTCTGAGCTATTCCAGAAGTAGTGAGGAGGCTTTTTTGGAGGCCTAGGCTTTTGCAAAAAGCTCCCGGGAGCTTGTATATCCATTTTCGGATC
TGATCAGCACGTGATGAAAAAGCCTGAACTCACCGCGACGTCTGTCGAGAAGTTTCTGATCGAAAAGTTCGACAGCGTTTCCGACCTGATGCAGCTCTCG
GAGGGCGAAGAATCTCGTGCTTTCAGCTTCGATGTAGGAGGGCGTGGATATGTCCTGCGGGTAAATAGCTGCGCCGATGGTTTCTACAAAGATCGTTATG
TTTATCGGCACTTTGCATCGGCCGCGCTCCCGATTCCGGAAGTGCTTGACATTGGGGAATTCAGCGAGAGCCTGACCTATTGCATCTCCCGCCGTGCACA
GGGTGTCACGTTGCAAGACCTGCCTGAAACCGAACTGCCCGCTGTTCTGCAGCCGGTCGCGGAGGCCATGGATGCGATCGCTGCGGCCGATCTTAGCCAG
ACGAGCGGGTTCGGCCCATTCGGACCGCAAGGAATCGGTCAATACACTACATGGCGTGATTTCATATGCGCGATTGCTGATCCCCATGTGTATCACTGGC
AAACTGTGATGGACGACACCGTCAGTGCGTCCGTCGCGCAGGCTCTCGATGAGCTGATGCTTTGGGCCGAGGACTGCCCCGAAGTCCGGCACCTCGTGCA
CGCGGATTTCGGCTCCAACAATGTCCTGACGGACAATGGCCGCATAACAGCGGTCATTGACTGGAGCGAGGCGATGTTCGGGGATTCCCAATACGAGGTC
GCCAACATCTTCTTCTGGAGGCCGTGGTTGGCTTGTATGGAGCAGCAGACGCGCTACTTCGAGCGGAGGCATCCGGAGCTTGCAGGATCGCCGCGGCTCC
GGGCGTATATGCTCCGCATTGGTCTTGACCAACTCTATCAGAGCTTGGTTGACGGCAATTTCGATGATGCAGCTTGGGCGCAGGGTCGATGCGACGCAAT
CGTCCGATCCGGAGCCGGGACTGTCGGGCGTACACAAATCGCCCGCAGAAGCGCGGCCGTCTGGACCGATGGCTGTGTAGAAGTACTCGCCGATAGTGGA
AACCGACGCCCCAGCACTCGTCCGAGGGCAAAGGAATAGCACGTGCTACGAGATTTCGATTCCACCGCCGCCTTCTATGAAAGGTTGGGCTTCGGAATCG
TTTTCCGGGACGCCGGCTGGATGATCCTCCAGCGCGGGGATCTCATGCTGGAGTTCTTCGCCCACCCCAACTTGTTTATTGCAGCTTATAATGGTTACAA
ATAAAGCAATAGCATCACAAATTTCACAAATAAAGCATTTTTTTCACTGCATTCTAGTTGTGGTTTGTCCAAACTCATCAATGTATCTTATCATGTCTGT
ATACCGTCGACCTCTAGCTAGAGCTTGGCGTAATCATGGTCATTACCAATGCTTAATCAGTGAGGCACCTATCTCAGCGATCTGTCTATTTCGTTCATCC
ATAGTTGCCTGACTCCCCGTCGTGTAGATAACTACGATACGGGAGGGCTTACCATCTGGCCCCAGCGCTGCGATGATACCGCGAGAACCACGCTCACCGG
CTCCGGATTTATCAGCAATAAACCAGCCAGCCGGAAGGGCCGAGCGCAGAAGTGGTCCTGCAACTTTATCCGCCTCCATCCAGTCTATTAATTGTTGCCG

GGAAGCTAGAGTAAGTAGTTCGCCAGTTAATAGTTTGCGCAACGTTGTTGCCATCGCTACAGGCATCGTGGTGTCACGCTCGTCGTTTGGTATGGCTTCA
TTCAGCTCCGGTTCCCAACGATCAAGGCGAGTTACATGATCCCCCATGTTGTGCAAAAAAGCGGTTAGCTCCTTCGGTCCTCCGATCGTTGTCAGAAGTA
AGTTGGCCGCAGTGTTATCACTCATGGTTATGGCAGCACTGCATAATTCTCTTACTGTCATGCCATCCGTAAGATGCTTTTCTGTGACTGGTGAGTACTC
AACCAAGTCATTCTGAGAATAGTGTATGCGGCGACCGAGTTGCTCTTGCCCGGCGTCAATACGGGATAATACCGCGCCACATAGCAGAACTTTAAAAGTG
CTCATCATTGGAAAACGTTCTTCGGGGCGAAAACTCTCAAGGATCTTACCGCTGTTGAGATCCAGTTCGATGTAACCCACTCGTGCACCCAACTGATCTT
CAGCATCTTTTACTTTCACCAGCGTTTCTGGGTGAGCAAAAACAGGAAGGCAAAATGCCGCAAAAAAGGGAATAAGGGCGACACGGAAATGTTGAATACT
CATATTCTTCCTTTTTCAATATTATTGAAGCATTTATCAGGGTTATTGTCTCATGAGCGGATACATATTTGAATGTATTTAGAAAAATAAACAAATAGGG
GTCAGTGTTACAACCAATTAACCAATTCTGAACATTATCGCGAGCCCATTTATACCTGAATATGGCTCATAACACCCCTTG

>TAL-PITCh vector for the *BLOS2* gene
CGCGATCACCAAGTATAAGGAGCCGCCGATCCTAGCCGCGAACCTGAACAATAGTCACCACCTGTTCCTGTAGCGGCCGCGACTCTAGATCATAATCAGC
CATACCACATTTGTAGAGGTTTTACTTGCTTTAAAAAACCTCCCACACCTCCCCCTGAACCTGAAACATAAAATGAATGCAATTGTTGTTGTTAACTTGT
TTATTGCAGCTTATAATGGTTACAAATAAAGCAATAGCATCACAAATTTCACAAATAAAGCATTTTTTTCACTGCATTCTAGTTGTGGTTTGTCCAAACT
CATCAATGTATCTTAAAGCTTATCGATACGCGTACAGATCTCAAAGAGGTCCGACGCGTATGTGCCGCAATATATATGACCCACTTTTATGCTTCAAACT
ATTTTTTACTGATGAGATAATTTCGGAAATTGTAAAATGGACAAATGCTGAGATATCATTGAAACGTCGGGAATCTATGACAGGTGCTACATTTCGTGAC
ACGAATGAAGATGAAATCTATGCTTTCTTTGGTATTCTGGTAATGACAGCAGTGAGAAAAGATAACCACATGTCCACAGATGACCTCTTTGATCGATCTT
TGTCAATGGTGTACGTCTCTGTAATGAGTCGTGATCGTTTTGATTTTTTGATACGATGTCTTAGAATGGATGACAAAAGTATACGGCCCACACTTCGAGA
AAACGATGTATTTACTCCTGTTAGAAAAATATGGGATCTCTTTATCCATCAGTGCATACAAAATTACACTCCAGGGGCTCATTTGACCATAGATGAACAG
TTACTTGGTTTTAGAGGACGGTGTCCGTTTAGGATGTATATCCCAAACAAGCCAAGTAAGTATGGAATAAAAATCCTCATGATGTGTGACAGTGGTACGA
AGTATATGATAAATGGAATGCCTTATTTGGGAAGAGGAACACAGACCAACGGAGTACCACTCGGTGAATACTACGTGAAGGAGTTATCAAAGCCTGTGCA
CGGTAGTTGTCGTAATATTACGTGTGACAATTGGTTCACCTCAATCCCTTTGGCAAAAACTTACTACAAGAACCGTATAAGGCCGCTCTAGCCCATGGC
TCAGTTCGCTTTAAATATCGATAGTCTTTAAAAAAAAATTATGATGTGAAATCTGAAAAGGTTTGCGCACTATGCGCTCATGCAATTTCAACTTTAATCA
ATAGCCCTAAAGCAGTGCTGCCAACAAATAAAAAAATATTTATTGTAAGTGAGATAACTAAATTTGTCAGAATTCATCGCTTCTTTGCTATTTTTTATTA
TGGTTTGTGTTTAATTATATTTTAAACGTGTTGATGAAATAGTAAGATAATTAGCATTAAATTTACACCACTTTCGTGAAGGTTTTCTGGCTAATCGTTT
CAGTTTGTGTGGCATAAAACTATGGGCTATAACTATTGCTGGTGAGACTTTAACAGCTTCATAAGGACGGGTGGATTAACTCAAAAACACAGCTTGGAGG
GAAAGAGTTTGCTAATAGCCGCCAGAGTGCCTCCAAAAGGGGTTTAATAGTACTAGGACAGTTACTTTACGAGTGCATCTACTCCGTGTCGAGAAGCTTA
GAGCTCAGTATCACATACAAATCGTCGACGAGTTAGCTGAACCATGCTTTTATTGCTCTTGGCGTTAAAATTAGTAGTGATAATGAGAACGTATCAGGTG
GATCTTGCATTATTTGAAAGATGAGACTAAAGATCTGTAGGTTGTGCGAGAGAATGTCGCGAGGCAAAAATAGTTTCCTCTACCTGTAGATAACGAGAAC
ACAAATTTTATACAGGGTGTATAGGAGAAGACGAGGAACAATTATTTTAATGATTTCTGACATCTAAATGAACAATAAGATTTTACTTTTGATGAACAAA
ACAAGTAACTCACATCTTAAACTGAACGTTACTACGTTCTGGTTTGCTATTGTAATTTTAATCAATGTTTTCTCCAATCTGCATTATGATACAATAAAAG
CTCATTATTCGCGTTTCCTTCACTCGCGTTTTCACTATTCGCAGATTAAAATGTGTCCCAATTCCTATTTTCGCAATTAAAGATCTCTTTATTCGCGGAT
CTAATCGATACATAGACATTGATAAAAAGAAATCCAATATTAAGTATTCAACTGAATATCCTTTATAATCGCGCTATAATCACGGTTTATCTATAAAGCC
TTGTTCGGACTAGGCGAGTATTTTAGTCGAGTACCGAGTAATTTAGTAGCTAAATGTGTCCGAGCACCAAAAGTTTAGTCGAGTAGGTTAGTAAATAATT
TAGTAAAGCCAATCCATTTTGTTCTATTTTATCGGGCGAGTAGCGCCTCCCACCACGCGTCCTAGCTCACTGGCTCACTGACTAAACAAGTCCGAACCTG
TTGATTAGTCGAGTTCATTAGTGGCGCTTCTCAAATGGAACTTTCGGACATTCTTAAACTATCGAAGTATTCTTTGGGATCTCTTCCAATGCTAATTCTT
TCAGAAGACATTTATCTCTTCAATCGATCCATTTTCGTACCTATAACTTATTTTTCATAATCACAGAAATTTGCTCGTCCACCTTTTCTAAAATAGCCAT
TACGATTAGCTTAATCAGTTTATTCACTCTATTGCGTTGCAGTCTGTTCATCCTGCAAAACACCACTCACTTGTGTACTCTACTGGACCGTAATGCGAAC
GATTTCTGTGCAGTAGCGAGTAGTTTAGCCACTAAATTACTCGGTACTCGACTAAAATACTCGCCTAGTCCGAACAAGGCTTTAGCGACATATTTACGTA
ACGTATACACCCCGATAAAGAGATTTACTGTCCAAAAAAAACGTCAAAAAATGGAACAAATTATGTGCTTTTTATTTATTGGTATTACTAATTTCGTTAA
GAATTTCATCGGAAGCAGCCAGCGCCGAATGCGGTGAATAGTAAAACTATAAGAAGGAAGCGGTTGGAATGATCTGGGTAAAATGTTATTTCTTACGAAT
TACGATGATTCTTTGATTAAATTTGGAAAAGTTTTGTGGTCAAATTATTTAACATTCATCTTGTATCATTTATTGACTCTAATTTCAACTAAGTTATTTC
TGTAATGTTAAAATTTCGATGCTTTTCTAGAATATTCAGGAATGTTCCAGATATTCTTGTGTGTAGATCGTATTGCCACCTTGTGCCAAATAGCGGCACT
AAAATAATTCGGCAGAGCTATACAACATTTGGTAATTTTATTTATTTTGCTATTTTCCCCAGTTTTTTAAATTACTGTGTTTTTAACTAGCAATTACATT
TAATTGGATGCAATTTACACTGCCTTAATAATTTTTTTTTAGTTGTAATTTGGTTTCTTACGTTTATGCTGTATATTTTTAGTGATCTGCAAAATGCGAA
ACACTGAACAGCTTAGGAAAATTTAAAATGTGAGATGTTTCTTTTACTTAAATAAATACTTAAAATATAACAGAGAAAAAACATTTTTAACTAGTCTGGC
ATATTTTAGTGAATCTATTGATGAAAATGAACGAAATATGTAATTACCTTTATAATTTCTTGAATTATTTTAAGGAAATATATTTTTCCTAACACTTTAA
ACTTCAAAGCGGGTGATGCAATAATAACTAACACAAAAAGAAACTTGCAGTGTTAAGTAAATGATTTTTCTCTTCGTTTTATGGCCTCCCAAAATGAAAT
AATTGCCAATTAATTTGAAATGATTAATTATTTTTATTTAATATTGTTTCGGTTTGTATTGATGGTAAAATGTTGATGCTCTCTGACGGAAATAGCGTCA
GAATCGAGAAACTTCTACTGATTCATAGATGTCGCTTGCTAGAGGAAAGTATAAAAACGAATTTACACACCGCGCCCGGCGTTATTTGAACTAAGAGAAG
TACGGAGACTAACGTTTGATACTTTGCGCTTTGAAACACGTGTGTTAAAAACCCTCTAGTCATTTTGTGTGAATTAAGCTAGTATGGTGAGCAAGGGCGA
GGAGCTGTTCACCGGGGTGGTGCCCATCCTGGTCGAGCTGGACGGCGACGTAAACGGCCACAAGTTCAGCGTGTCCGGCGAGGGCGAGGGCGATGCCACC
TACGGCAAGCTGACCCTGAAGTTCATCTGCACCACCGGCAAGCTGCCCGTGCCCTGGCCCACCCTCGTGACCACCCTGACCTACGGCGTGCAGTGCTTCA
GCCGCTACCCCGACCACATGAAGCAGCACGACTTCTTCAAGTCCGCCATGCCCGAAGGCTACGTCCAGGAGCGCACCATCTTCTTCAAGGACGACGGCAA
CTACAAGACCCGCGCCGAGGTGAAGTTCGAGGGCGACACCCTGGTGAACCGCATCGAGCTGAAGGGCATCGACTTCAAGGAGGACGGCAACATCCTGGGG
CACAAGCTGGAGTACAACTACAACAGCCACAACGTCTATATCATGGCCGACAAGCAGAAGAACGGCATCAAGGTGAACTTCAAGATCCGCCACAACATCG
AGGACGGCAGCGTGCAGCTCGCCGACCACTACCAGCAGAACACCCCCATCGGCGACGGCCCCGTGCTGCTGCCCGACAACCACTACCTGAGCACCCAGTC
CGCCCTGAGCAAAGACCCCAACGAGAAGCGCGATCACATGGTCCTGCTGGAGTTCGTGACCGCCGCCGGGATCACTCTCGGCATGGACGAGCTGTACaAG
TAATCTAGGTCAGCCATACCACATTTGTAGAGGTTTTACTTGCTTTAAAAAACCTCCCACACCTCCCCCTGAACCTGAAACATAAAATGAATGCAATTGT
TGTTGTTAACTTGTTTATTGCAGCTTATAATGGTTACAAATAAAGCAATAGCATCACAAATTTCACAAATAAAGCATTTTTTTCACTGCATTCTAGTTGT
GGTTTGTCCAAACTCATCAATGTATCTTAAGGCGTAAATTGTAAGCGTTAATATTTTGTTAAAATTCGCGTTAAATTTTTGTTAAATCAGCTCATTTTTT
AACCAATAGGCCGAAATCGGCAAAATCCCTTATAAATCAAAAGAATAGACCGAGATAGGGTTGAGTGTTGTTCCAGGGCGCGCCATAAAAGTTTTGTTAC
TTTATAGAAGAAATTTTGAGTTTTTGTTTTTTTTTAATAAATAAATAAACATAAATAAATTGTTTGTTGAATTTATTATTAGTATGTAAGTGTAAATATA
ATAAAACTTAATATCTATTCAAATTAATAAATAAACCTCGATATACAGACCGATAAAACACATGCGTCAATTTTACGCATGATTATCTTTAACGTACGTC
ACAATATGATTATCTTTCTAGGGTTAAATAATAGTTTCTAATTTTTTTATTATTCAGCCTGCTGTCGTGAATACCGTATATCTCAACGCTGTCTGTGAGA
TTGTCGTATTCTAGCCTTTTTAGTTTTTCGCTCATCGACTTGATATTGTCCGACACATTTTCGTCGATTTGCGTTTTGATCAAAGACTTGAGCAGAGACA
CGTTAATCAACTGTTCAAATTGATCCATATTAACGATATCAACCCGATGCGTATATGGTGCGTAAAATATATTTTTTAACCCTCTTATACTTTGCACTCT
GCGTTAATACGCGTTCGTGTACAGACGTAATCATGTTTTCTTTTTTGGATAAAACTCCTACTGAGTTTGACCTCATATTAGACCCTCACAAGTTGCAAAA
CGTGGCATTTTTTACCAATGAAGAATTTAAAGTTATTTTAAAAAATTTCATCACAGATTTAAAGAAGAACCAAAAATTAAATTATTTAATCGACCAGTTA
ATCAACGTGTACACAGAGCGCAAAAAACACGCAGCCCGACGTGTTGGCTAAAATTATTAAATCAACTTGTGTTATAGTCACGATTTGCCGTCCAACGTGT
TCCTCAAAAAGTTGAAGACCAACAAGTTTACGGACACTAGTTAATTATTTGATTTTGCCCCACTTCATTTTGTGGGATCACAATTTTGTTATATTTTAAA
CAAAGCTTGGCACTGGCCGTCGTTTTACAACGTCGTGACTGGGAAAACCCTGGCGTTACCCAACTTAATCGCCTTGCAGCACATCCCCCTTTCGCCAGCT
GGCGTAATAGCGAAGAGGCCCGCACCGATCGCCCTTCCCAACAGTTGCGCAGCCTGAATGGCGAATGGCGCCTGATGCGGTATTTTCTCCTTACGCATCT
GTGCGGTATTTCACACCGCATATGGTGCACTCTCAGTACAATCTGCTCTGATGCCGCATAGTTAAGCCAGCCCCGACACCCGCCAACACCCGCTGACGCG
CCCTGACGGGCTTGTCTGCTCCCGGCATCCGCTTACAGACAAGCTGTGACCGTCTCCGGGAGCTGCATGTGTCAGAGGTTTTCACCGTCATCACCGAAAC
GCGCGAGACGAAAGGGCCTCGTGATACGCCTATTTTTATAGGTTAATGTCATGATAATAATGGTTTCTTAGACGTCAGGTGGCACTTTTCGGGGAAATGT
GCGCGGAACCCCTATTTGTTTATTTTTCTAAATACATTCAAATATGTATCCGCTCATGAGACAATAACCCTGATAAATGCTTCAATAATATTGAAAAAGG
AAGAGTATGAGTATTCAACATTTCCGTGTCGCCCTTATTCCCTTTTTTGCGGCATTTTGCCTTCCTGTTTTTGCTCACCCAGAAACGCTGGTGAAAGTAA
AAGATGCTGAAGATCAGTTGGGTGCACGAGTGGGTTACATCGAACTGGATCTCAACAGCGGTAAGATCCTTGAGAGTTTTCGCCCCGAAGAACGTTTTCC
AATGATGAGCACTTTTAAAGTTCTGCTATGTGGCGCGGTATTATCCCGTATTGACGCCGGGCAAGAGCAACTCGGTCGCCGCATACACTATTCTCAGAAT
GACTTGGTTGAGTACTCACCAGTCACAGAAAAGCATCTTACGGATGGCATGACAGTAAGAGAATTATGCAGTGCTGCCATAACCATGAGTGATAACACTG
CGGCCAACTTACTTCTGACAACGATCGGAGGACCGAAGGAGCTAACCGCTTTTTTGCACAACATGGGGGATCATGTAACTCGCCTTGATCGTTGGGAACC
GGAGCTGAATGAAGCCATACCAAACGACGAGCGTGACACCACGATGCCTGTAGCAATGGCAACAACGTTGCGCAAACTATTAACTGGCGAACTACTTACT
CTAGCTTCCCGGCAACAATTAATAGACTGGATGGAGGCGGATAAAGTTGCAGGACCACTTCTGCGCTCGGCCCTTCCGGCTGGCTGGTTTATTGCTGATA
AATCTGGAGCCGGTGAGCGTGGGTCTCGCGGTATCATTGCAGCACTGGGGCCAGATGGTAAGCCCTCCCGTATCGTAGTTATCTACACGACGGGGAGTCA
GGCAACTATGGATGAACGAAATAGACAGATCGCTGAGATAGGTGCCTCACTGATTAAGCATTGGTAACTGTCAGACCAAGTTTACTCATATATACTTTAG
ATTGATTTAAAACTTCATTTTTAATTTAAAAGGATCTAGGTGAAGATCCTTTTTGATAATCTCATGACCAAAATCCCTTAACGTGAGTTTTCGTTCCACT
GAGCGTCAGACCCCGTAGAAAAGATCAAAGGATCTTCTTGAGATCCTTTTTTTCTGCGCGTAATCTGCTGCTTGCAAACAAAAAAACCACCGCTACCAGC

GGTGGTTTGTTTGCCGGATCAAGAGCTACCAACTCTTTTTCCGAAGGTAACTGGCTTCAGCAGAGCGCAGATACCAAATACTGTCCTTCTAGTGTAGCCG
TAGTTAGGCCACCACTTCAAGAACTCTGTAGCACCGCCTACATACCTCGCTCTGCTAATCCTGTTACCAGTGGCTGCTGCCAGTGGCGATAAGTCGTGTC
TTACCGGGTTGGACTCAAGACGATAGTTACCGGATAAGGCGCAGCGGTCGGGCTGAACGGGGGGTTCGTGCACACAGCCCAGCTTGGAGCGAACGACCTA
CACCGAACTGAGATACCTACAGCGTGAGCATTGAGAAAGCGCCACGCTTCCCGAAGGGAGAAAGGCGGACAGGTATCCGGTAAGCGGCAGGGTCGGAACA
GGAGAGCGCACGAGGGAGCTTCCAGGGGGAAACGCCTGGTATCTTTATAGTCCTGTCGGGTTTCGCCACCTCTGACTTGAGCGTCGATTTTTGTGATGCT
CGTCAGGGGGGCGGAGCCTATGGAAAAACGCCAGCAACGCGGCCTTTTTACGGTTCCTGGCCTTTTGCTGGCCTTTTGCTCACATGTTCTTTCCTGCGTT
ATCCCCTGATTCTGTGGATAACCGTATTACCGCCTTTGAGTGAGCTGATACCGCTCGCCGCAGCCGAACGACCGAGCGCAGCGAGTCAGTGAGCGAGGAA
GCGGAAGAGCGCCCAATACGCAAACCGCCTCTCCCCGCGCGTTGGCCGATTCATTAATGCAGCTGGCACGACAGGTTTCCCGACTGGAAAGCGGGCAGTG
AGCGCAACGCAATTAATGTGAGTTAGCTCACTCATTAGGCACCCCAGGCTTTACACTTTATGCTTCCGGCTCGTATGTTGTGTGGAATTGTGAGCGGATA
ACAATTTCACACAGGAAACAGCTATGACCATGATTACGAATTGATCCTCTAGCTAGAGTCGACGCTCGCGCGACTTGGTTTGCCATTCTTTAGCGCGCGT
CGCGTCACACAGCTTGGCCACAATGTGGTTTTTGTCAAACGAAGATTCTATGACGTGTTTAAAGTTTAGGTCGAGTAAAGCGCAAATCTTTTTTAACCCT
AGAAAGATAGTCTGCGTAAAATTGACGCATGCATTCTTGAAATATTGCTCTCTCTTTCTAAATAGCGCGAATCCGTCGCTGTGCATTTAGGACATCTCAG
TCGCCGCTTGGAGCTCCCGTGAGGCGTGCTTGTCAATGCGGTAAGTGTCACTGATTTTGAACTATAACGACCGCGTGAGTCAAAATGACGCATGATTATC
TTTTACGTGACTTTTAAGATTTAACTCATACGATAATTATATTGTTATTTCATGTTCTACTTACGTGATAACTTATTATATATATATTTTCTTGTTATAG
ATATCAAGCTTATCGATACCGTCGACCTCGAGGGGGGGCCCGGTACCCAAAGATCTAATTCGAG

>TAL-PITCh vector for the *no29* gene
TGGGTATCAGTGCAGCAGACAGGGCAAGACTGTAGATCGGTTTTACTATGAGCGGCTTTATATCAAGCACAGCGGCACAAGGGCCGCCGAGCCCAGCAAT
TGAGTCCTATTTATTTGGCTGTGAATTGTCATCAAAAACAAAACAATACACTTTTGAGGTAAATGAAGAGGATGATGCTGTGCACCTTGTGTGCTTACAG
ACTATTAGTCTTGGGGCAGGAGCTAAGGATGAACACAATGTAGTGGAAGTGACTGCGCCTAATTATCAAAATAAGGAAGTTACAGTTCCTTTGGCGAACC
TTAAACTTTCATGTCAGCCAATGGTAAATGTGGGATATTTTGAGATTGAGGCACCCGTGACCTTTCGTTTAACATCTGGTTCTGGTCCAGTTTTTATCTC
CGGTCGTCATTACGTTGTAGCCAGTGATGATGAAGACCTGTCTGGTTCAGAGGAAGAGATGGAAGATGATGGCGAGGAGGAGGAGGAGGATGATGATGAT
GATGATGACGACGATGATGATGATGATGATGAAGAAGAAATTACTCCAATAAAACCAGCTAAGAAACCCCTCAAAACTCTATCCCGTACCTTCGGCGGCG
GATCCATCGCCACCATGGTGAGCAAGGGCGAGGAGCTGTTCACCGGGGTGGTGCCCATCCTGGTCGAGCTGGACGGCGACGTAAACGGCCACAAGTTCAG
CGTGTCCGGCGAGGGCGAGGGCGATGCCACCTACGGCAAGCTGACCCTGAAGTTCATCTGCACCACCGGCAAGCTGCCCGTGCCCTGGCCCACCCTCGTG
ACCACCCTGACCTACGGCGTGCAGTGCTTCAGCCGCTACCCCGACCACATGAAGCAGCACGACTTCTTCAAGTCCGCCATGCCCGAAGGCTACGTCCAGG
AGCGCACCATCTTCTTCAAGGACGACGGCAACTACAAGACCCGCGCCGAGGTGAAGTTCGAGGGCGACACCCTGGTGAACCGCATCGAGCTGAAGGGCAT
CGACTTCAAGGAGGACGGCAACATCCTGGGGCACAAGCTGGAGTACAACTACAACAGCCACAACGTCTATATCATGGCCGACAAGCAGAAGAACGGCATC
AAGGTGAACTTCAAGATCCGCCACAACATCGAGGACGGCAGCGTGCAGCTCGCCGACCACTACCAGCAGAACACCCCCATCGGCGACGGCCCCGTGCTGC
TGCCCGACAACCACTACCTGAGCACCCAGTCCGCCCTGAGCAAAGACCCCAACGAGAAGCGCGATCACATGGTCCTGCTGGAGTTCGTGACCGCCGCCGG
GATCACTCTCGGCATGGACGAGCTGTACAAGTAAAGCGGCCGCGACTCTAGATCATAATCAGCCATACCACATTTGTAGAGGTTTTACTTGCTTTAAAAA
ACCTCCCACACCTCCCCCTGAACCTGAAACATAAAATGAATGCAATTGTTGTTGTTAACTTGTTTATTGCAGCTTATAATGGTTACAAATAAAGCAATAG
CATCACAAATTTCACAAATAAAGCATTTTTTTCACTGCATTCTAGTTGTGGTTTGTCCAAACTCATCAATGTATCTTAAGGCGTAAATTGTAAGCGTTAA
TATTTTGTTAAAATTCGCGTTAAATTTTTGTTAAATCAGCTCATTTTTTAACCAATAGGCCGAAATCGGCAAAATCCCTTATAAATCAAAAGAATAGACC
GAGATAGGGTTGAGTGTTGTTCCAGTTTGGAACAAGAGTCCACTATTAAAGAACGTGGACTCCAACGTCAAAGGGCGAAAAACCGTCTATCAGGGCGATG
GCCCACTACGTGAACCATCACCCTAATCAAGTTTTTTGGGGTCGAGGTGCCGTAAAGCACTAAATCGGAACCCTAAAGGGAGCCCCCGATTTAGAGCTTG
ACGGGGAAAGCCGGCGAACGTGGCGAGAAAGGAAGGGAAGAAAGCGAAAGGAGCGGGCGCTAGGGCGCTGGCAAGTGTAGCGGTCACGCTGCGCGTAACC
ACCACACCCGCCGCGCTTAATGCGCCGCTACAGGGCGCGTCAGGTGGCACTTTTCGGGGAAATGTGCGCGGAACCCCTATTTGTTTATTTTTCTAAATAC
ATTCAAATATGTATCCGCTCATGAGACAATAACCCTGATAAATGCTTCAATAATATTGAAAAAGGAAGAGTCCTGAGGCGGAAAGAACCAGCTGTGGAAT
GTGTGTCAGTTAGGGTGTGGAAAGTCCCCAGGCTCCCCAGCAGGCAGAAGTATGCAAAGCATGCATCTCAATTAGTCAGCAACCAGGTGTGGAAAGTCCC
CAGGCTCCCCAGCAGGCAGAAGTATGCAAAGCATGCATCTCAATTAGTCAGCAACCATAGTCCCGCCCCTAACTCCGCCCATCCCGCCCCTAACTCCGCC
CAGTTCCGCCCATTCTCCGCCCCATGGCTGACTAATTTTTTTTATTTATGCAGAGGCCGAGGCCGCCTCGGCCTCTGAGCTATTCCAGAAGTAGTGAGGA
GGCTTTTTTGGAGGCCTAGGCTTTTGCAAAGATCGATCAAGAGACAGGATGAGGATCGTTTCGCATGATTGAACAAGATGGATTGCACGCAGGTTCTCCG
GCCGCTTGGGTGGAGAGGCTATTCGGCTATGACTGGGCACAACAGACAATCGGCTGCTCTGATGCCGCCGTGTTCCGGCTGTCAGCGCAGGGGCGCCCGG
TTCTTTTTGTCAAGACCGACCTGTCCGGTGCCCTGAATGAACTGCAAGACGAGGCAGCGCGGCTATCGTGGCTGGCCACGACGGGCGTTCCTTGCGCAGC
TGTGCTCGACGTTGTCACTGAAGCGGGAAGGGACTGGCTGCTATTGGGCGAAGTGCCGGGGCAGGATCTCCTGTCATCTCACCTTGCTCCTGCCGAGAAA
GTATCCATCATGGCTGATGCAATGCGGCGGCTGCATACGCTTGATCCGGCTACCTGCCCATTCGACCACCAAGCGAAACATCGCATCGAGCGAGCACGTA
CTCGGATGGAAGCCGGTCTTGTCGATCAGGATGATCTGGACGAAGAGCATCAGGGGCTCGCGCCAGCCGAACTGTTCGCCAGGCTCAAGGCGAGCATGCC
CGACGGCGAGGATCTCGTCGTGACCCATGGCGATGCCTGCTTGCCGAATATCATGGTGGAAAATGGCCGCTTTTCTGGATTCATCGACTGTGGCCGGCTG
GGTGTGGCGGACCGCTATCAGGACATAGCGTTGGCTACCCGTGATATTGCTGAAGAGCTTGGCGGCGAATGGGCTGACCGCTTCCTCGTGCTTTACGGTA
TCGCCGCTCCCGATTCGCAGCGCATCGCCTTCTATCGCCTTCTTGACGAGTTCTTCTGAGCGGGACTCTGGGGTTCGAAATGACCGACCAAGCGACGCCC
AACCTGCCATCACGAGATTTCGATTCCACCGCCGCCTTCTATGAAAGGTTGGGCTTCGGAATCGTTTTCCGGGACGCCGGCTGGATGATCCTCCAGCGCG
GGGATCTCATGCTGGAGTTCTTCGCCCACCCTAGGGGGAGGCTAACTGAAACACGGAAGGAGACAATACCGGAAGGAACCCGCGCTATGACGGCAATAAA
AAGACAGAATAAAACGCACGGTGTTGGGTCGTTTGTTCATAAACGCGGGGTTCGGTCCCAGGGCTGGCACTCTGTCGATACCCCACCGAGACCCCATTGG
GGCCAATACGCCCGCGTTTCTTCCTTTTCCCCACCCCACCCCCCAAGTTCGGGTGAAGGCCCAGGGCTCGCAGCCAACGTCGGGGCGGCAGGCCCTGCCA
TAGCCTCAGGTTACTCATATATACTTTAGATTGATTTAAAACTTCATTTTTAATTTAAAAGGATCTAGGTGAAGATCCTTTTTGATAATCTCATGACCAA
AATCCCTTAACGTGAGTTTTCGTTCCACTGAGCGTCAGACCCCGTAGAAAAGATCAAAGGATCTTCTTGAGATCCTTTTTTTCTGCGCGTAATCTGCTGC
TTGCAAACAAAAAAACCACCGCTACCAGCGGTGGTTTGTTTGCCGGATCAAGAGCTACCAACTCTTTTTCCGAAGGTAACTGGCTTCAGCAGAGCGCAGA
TACCAAATACTGTCCTTCTAGTGTAGCCGTAGTTAGGCCACCACTTCAAGAACTCTGTAGCACCGCCTACATACCTCGCTCTGCTAATCCTGTTACCAGT
GGCTGCTGCCAGTGGCGATAAGTCGTGTCTTACCGGGTTGGACTCAAGACGATAGTTACCGGATAAGGCGCAGCGGTCGGGCTGAACGGGGGGTTCGTGC
ACACAGCCCAGCTTGGAGCGAACGACCTACACCGAACTGAGATACCTACAGCGTGAGCTATGAGAAAGCGCCACGCTTCCCGAAGGGAGAAAGGCGGACA
GGTATCCGGTAAGCGGCAGGGTCGGAACAGGAGAGCGCACGAGGGAGCTTCCAGGGGGAAACGCCTGGTATCTTTATAGTCCTGTCGGGTTTCGCCACCT
CTGACTTGAGCGTCGATTTTTGTGATGCTCGTCAGGGGGGCGGAGCCTATGGAAAAACGCCAGCAACGCGGCCTTTTTACGGTTCCTGGCCTTTTGCTGG
CCTTTTGCTCACATGTTCTTTCCTGCGTTATCCCCTGATTCTGTGGATAACCGTATTACCGCCATGCAT

>TAL-PITCh vector for the *fgk* gene
TCAAGAAGTAGAAGAGAAATATAACAAACTCAATACTGATAGAAGAGCATTATGGCCGGTCGCCACCATGGTGAGCAAGGGCGAGGAGCTGTTCACCGGG
GTGGTGCCCATCCTGGTCGAGCTGGACGGCGACGTAAACGGCCACAAGTTCAGCGTGTCCGGCGAGGGCGAGGGCGATGCCACCTACGGCAAGCTGACCC
TGAAGTTCATCTGCACCACCGGCAAGCTGCCCGTGCCCTGGCCCACCCTCGTGACCACCCTGACCTACGGCGTGCAGTGCTTCAGCCGCTACCCCGACCA
CATGAAGCAGCACGACTTCTTCAAGTCCGCCATGCCCGAAGGCTACGTCCAGGAGCGCACCATCTTCTTCAAGGACGACGGCAACTACAAGACCCGCGCC
GAGGTGAAGTTCGAGGGCGACACCCTGGTGAACCGCATCGAGCTGAAGGGCATCGACTTCAAGGAGGACGGCAACATCCTGGGGCACAAGCTGGAGTACA
ACTACAACAGCCACAACGTCTATATCATGGCCGACAAGCAGAAGAACGGCATCAAGGTGAACTTCAAGATCCGCCACAACATCGAGGACGGCAGCGTGCA
GCTCGCCGACCACTACCAGCAGAACACCCCCATCGGCGACGGCCCCGTGCTGCTGCCCGACAACCACTACCTGAGCACCCAGTCCGCCCTGAGCAAAGAC
CCCAACGAGAAGCGCGATCACATGGTCCTGCTGGAGTTCGTGACCGCCGCCGGGATCACTCTCGGCATGGACGAGCTGTACAAGTAAAGCGGCCGCGACT
CTAGAACTATAGTGAGTCGTATTACGTAGATCCAGACATGATAAGATACATTGATGAGTTTGGACAAACCACAACTAGAATGCAGTGAAAAAAATGCTTT
ATTTGTGAAATTTGTGATGCTATTGCTTTATTTGTAACCATTATAAGCTGCAATAAACAAGTTAACAACAACAATTGCATTCATTTTATGTTTCAGGTTC
AGGGGGAGGTGTGGGAGGTTTTTTAATTCGCGGCCGCGGCGCCAATGCATTGGGCCCGGTACCCAGCTTTTGTTCCCTTTAGTGAGGGTTAATTGCGCGC
TTGGCGTAATCATGGTCATAGCTGTTTCCTGTGTGAAATTGTTATCCGCTCACAATTCCACACAACATACGAGCCGGAAGCATAAAGTGTAAAGCCTGGG
GTGCCTAATGAGTGAGCTAACTCACATTAATTGCGTTGCGCTCACTGCCCGCTTTCCAGTCGGGAAACCTGTCGTGCCAGCTGCATTAATGAATCGGCCA
ACGCGCGGGGAGAGGCGGTTTGCGTATTGGGCGCTCTTCCGCTTCCTCGCTCACTGACTCGCTGCGCTCGGTCGTTCGGCTGCGGCGAGCGGTATCAGCT
CACTCAAAGGCGGTAATACGGTTATCCACAGAATCAGGGGATAACGCAGGAAAGAACATGTGAGCAAAAGGCCAGCAAAAGGCCAGGAACCGTAAAAAGG
CCGCGTTGCTGGCGTTTTTCCATAGGCTCCGCCCCCCTGACGAGCATCACAAAAATCGACGCTCAAGTCAGAGGTGGCGAAACCCGACAGGACTATAAAG
ATACCAGGCGTTTCCCCCTGGAAGCTCCCTCGTGCGCTCTCCTGTTCCGACCCTGCCGCTTACCGGATACCTGTCCGCCTTTCTCCCTTCGGGAAGCGTG
GCGCTTTCTCATAGCTCACGCTGTAGGTATCTCAGTTCGGTGTAGGTCGTTCGCTCCAAGCTGGGCTGTGTGCACGAACCCCCCGTTCAGCCCGACCGCT
GCGCCTTATCCGGTAACTATCGTCTTGAGTCCAACCCGGTAAGACACGACTTATCGCCACTGGCAGCAGCCACTGGTAACAGGATTAGCAGAGCGAGGTA
TGTAGGCGGTGCTACAGAGTTCTTGAAGTGGTGGCCTAACTACGGCTACACTAGAAGGACAGTATTTGGTATCTGCGCTCTGCTGAAGCCAGTTACCTTC

GGAAAAAGAGTTGGTAGCTCTTGATCCGGCAAACAAACCACCGCTGGTAGCGGTGGTTTTTTTGTTTGCAAGCAGCAGATTACGCGCAGAAAAAAAGGAT
CTCAAGAAGATCCTTTGATCTTTTCTACGGGGTCTGACGCTCAGTGGAACGAAAACTCACGTTAAGGGATTTTGGTCATGAGATTATCAAAAAGGATCTT
CACCTAGATCCTTTTAAATTAAAAATGAAGTTTTAAATCAATCTAAAGTATATATGAGTAAACTTGGTCTGACAGTTACCAATGCTTAATCAGTGAGGCA
CCTATCTCAGCGATCTGTCTATTTCGTTCATCCATAGTTGCCTGACTCCCCGTCGTGTAGATAACTACGATACGGGAGGGCTTACCATCTGGCCCCAGTG
CTGCAATGATACCGCGAGACCCACGCTCACCGGCTCCAGATTTATCAGCAATAAACCAGCCAGCCGGAAGGGCCGAGCGCAGAAGTGGTCCTGCAACTTT
ATCCGCCTCCATCCAGTCTATTAATTGTTGCCGGGAAGCTAGAGTAAGTAGTTCGCCAGTTAATAGTTTGCGCAACGTTGTTGCCATTGCTACAGGCATC
GTGGTGTCACGCTCGTCGTTTGGTATGGCTTCATTCAGCTCCGGTTCCCAACGATCAAGGCGAGTTACATGATCCCCCATGTTGTGCAAAAAAGCGGTTA
GCTCCTTCGGTCCTCCGATCGTTGTCAGAAGTAAGTTGGCCGCAGTGTTATCACTCATGGTTATGGCAGCACTGCATAATTCTCTTACTGTCATGCCATC
CGTAAGATGCTTTTCTGTGACTGGTGAGTACTCAACCAAGTCATTCTGAGAATAGTGTATGCGGCGACCGAGTTGCTCTTGCCCGGCGTCAATACGGGAT
AATACCGCGCCACATAGCAGAACTTTAAAAGTGCTCATCATTGGAAAACGTTCTTCGGGGCGAAAACTCTCAAGGATCTTACCGCTGTTGAGATCCAGTT
CGATGTAACCCACTCGTGCACCCAACTGATCTTCAGCATCTTTTACTTTCACCAGCGTTTCTGGGTGAGCAAAAACAGGAAGGCAAAATGCCGCAAAAAA
GGGAATAAGGGCGACACGGAAATGTTGAATACTCATACTCTTCCTTTTTCAATATTATTGAAGCATTTATCAGGGTTATTGTCTCATGAGCGGATACATA
TTTGAATGTATTTAGAAAAATAAACAAATAGGGGTTCCGCGCACATTTCCCCGAAAAGTGCCACCTAAATTGTAAGCGTTAATATTTTGTTAAAATTCGC
GTTAAATTTTTGTTAAATCAGCTCATTTTTTAACCAATAGGCCGAAATCGGCAAAATCCCTTATAAATCAAAAGAATAGACCGAGATAGGGTTGAGTGTT
GTTCCAGTTTGGAACAAGAGTCCACTATTAAAGAACGTGGACTCCAACGTCAAAGGGCGAAAAACCGTCTATCAGGGCGATGGCCCACTACGTGAACCAT
CACCCTAATCAAGTTTTTTGGGGTCGAGGTGCCGTAAAGCACTAAATCGGAACCCTAAAGGGAGCCCCCGATTTAGAGCTTGACGGGGAAAGCCGGCGAA
CGTGGCGAGAAAGGAAGGGAAGAAAGCGAAAGGAGCGGGCGCTAGGGCGCTGGCAAGTGTAGCGGTCACGCTGCGCGTAACCACCACACCCGCCGCGCTT
AATGCGCCGCTACAGGGCGCGTCCCATTCGCCATTCAGGCTGCGCAACTGTTGGGAAGGGCGATCGGTGCGGGCCTCTTCGCTATTACGCCAGTCGACCA
TAGCC

Supplementary Figure 12 Full plasmid sequences of the TAL-PITCh, CRIS-PITCh, and HR vectors. Green, purple, blue and red letters indicate the coding sequences of mNeonGreen/EGFP, 2A peptide, the puromycin resistance gene and no29, respectively. Light blue letters indicate the *hsp90* promoter. Target sites for TALENs and CRISPR/Cas9 are underlined.

Supplementary Table 1. TAL-PITCh and CRIS-PITCh efficiencies in human cells.

	Gene	Cell line	PCR positive clone/total clone			Knockin with precise 5′ junction	Knockin with precise 3′ junction
			5′ junction	3′ junction	Both junctions		
TAL-PITCh	*FBL*	HEK293T	5/6	4/6	4/6	4/4 (100%)	4/4 (100%)
	ACTB	HeLa	6/6	4/6	4/6	3/4 (75%)	1/4 (25%)
CRIS-PITCh	*FBL*	HEK293T	5/5	4/5	4/5	2/4 (50%)	0/4 (0%)

Supplementary Table 2. Summary of silkworm experiments.

G_0		
Eggs injected	Hatched larvae	Adults crossed with non-injected individuals
181	62	35

G_1					
Batches with EGFP-positive embryos	Total number of EGFP-positive embryos	Genotyped individuals	Knockin into *BLOS2* locus	Knockin with precise 5′ junction	Knockin with precise 3′ junction
8	92	36	6	6 (100%)	4 (67%)

Supplementary Table 3. Summary of the off-target analyses for TAL-PITCh.

Name	Sequence*	TALEN score**	Coordinate	Genomic region	Mutation
On-target	5′-TCCTGACAGCGCTGAACTtcagttcttcaccttGGGGGGTGGCCTGTGAGA-3′<br>3′-AGGACTGTCGCGACTTGAagtcaagaagtggaaCCCCCCACCGGACACTCT-5′	100	chr19:40325160 -40325210	Exon	
TAL-OT1	5′-TCTCCCAGGCCACCCCTCggtcccagagtctcctcccCGTCCAGAGCTCTCAGGA-3′<br>3′-AGAGGGTCCGGTGGGGAGccagggtctcagaggagggGCAGGTCTCGAGAGTCCT-5′	61.72	chr5:42953443-42953497	Intergenic	Not detected
TAL-OT2	5′-TCTCACATGCCACCCCACtcaatgggtggtgcttCTTTGAGCACTGTAAGGT-3′<br>3′-AGAGTGTACGGTGGGGTGagttacccaccacgaaGAAACTCGTGACATTCCA-5′	58.44	chr10:98256567 -98256618	Intron	Not detected
TAL-OT3	5′-TCCTGACTGCACAGAACTggacaagagctagttGCTGGGTGGGTTGGGGA-3′<br>3′-AGGACTGACGTGTCTTGAcctgttctcgatcaaCGACCCACCCAACCCCCT-5′	56.82	chr16:70751469 -70751519	Intron	Not detected
TAL-OT4	5′-TCCTGACCTCCCTGAACTccacccaccttggcctcccaaAGTGCTGGGATTACAGGA-3′<br>3′-AGGACTGGAGGGACTTGAggtgggtggaaccggagggttTCACGACCCTAATGTCCT-5′	56.56	chr5:61711213-61711269	Intron	Not detected
TAL-OT5	5′-TCACAAAAACACTGAACTtgagcaaatattgtcAGTTCTGGGGTGGCAGGA-3′<br>3′-AGTGTTTTTGTGACTTGAactcgtttataacagTCAAGACCCCACCGTCCT-5′	56.3	chr3:178104774 -178104824	Intergenic	Not detected
TAL-OT6	5′-TCTCACAGGCCACCACCAaggtgtcagccaggcCTGGGGTCTCCTTTGATA-3′<br>3′-AGAGTGTCCGGTGGTGGTtccacagtcggtccgGACCCCAGAGGAAACTAT-5′	56.11	chr7:153319981 -153320031	Intergenic	Not detected

*Red and blue letters indicate left and right TALEN target sequences, respectively. Mismatches are underlined.

**TALEN scores were calculated by the PROGNOS tool (http://baolab.bme.gatech.edu/cgi-bin/prognos/prognos.cgi).

Supplementary Table 4. Summary of the off-target analyses for CRIS-PITCh.

Name	Sequence*	Score**	Coordinate	Strand	Mutation
On-target (gRNA on the genome)	CTCTCACAGGCCACCCCCCAAGG				
CRIS-OT1A	CCCTCCCAGGCCACCCCCCAAGG	3.6	chr18:46206217	-	Not detected
CRIS-OT2A	TGATCACAGGCCACCCCCCATAG	2.3	chr6:30796131	-	Not detected
CRIS-OT3A	CTCTCTCAGGCCACCCTCCAGAG	2.2	chr11:76938544	+	Not detected
On-target (gRNA at the 5' junction)	TGGATCCGTGGGGTGGCCCCGGG				
CRIS-OT1B	TGGCTCCGTGGGGTGGCCGCTGG	4.3	chr10:29645541	-	Not detected
CRIS-OT2B	TTGCTCCTTGGGGTGGCCCCAGG	2.5	chr22:38619312	+	Not detected
CRIS-OT3B	TGGATCTGGGGGGTGGCCCCTGG	2.3	chr3:133419488	+	Not detected
On-target (gRNA at the 3' junction)	GGTGCCTGACCAAGGTGCCCGGG				
CRIS-OT1C	TGTGTCTGAGCAAGGTGCCCAGG	2.5	chr7:29849668	+	Not detected
CRIS-OT2C	GCTGGCTGGCCAAGGTGCCCGAG	1.6	chr15:61098465	+	Not detected
CRIS-OT3C	GGAGCCTGGGCAAGGTGCCCAAG	1.4	chr17:79974949	+	Not detected

* Red letters indicate PAM sequence. Mismatches are underlined.

**Scores were calculated by the CRISPR design tool (http://crispr.mit.edu/).

Supplementary Table 5. Oligonucleotides for gRNA templates.

Gene	Target locus	Sense oligonucleotide (5′->3′)	Antisense oligonucleotide (5′->3′)
FBL	Genome	caccgctctcacaggccacccccca	aaactggggggtggcctgtgagagc
	5' junction	caccgtggatccgtggggtggcccc	aaacggggccaccccacggatccac
	3' junction	caccggtgcctgaccaaggtgccc	aaacgggcaccttggtcaggcacc

Supplementary Table 6. Primers for PCR at the on-target sites.

	Gene	Locus	Forward primer (5′->3′)	Reverse primer (5′->3′)
TAL-PITCh in human cells	*FBL*	5' junction	acaccaagacagacatctctgtcccttg	atccgtatccaatgtggggaac
		3' junction	gcccttaattgtgagcggataac	tcagcaggtcaaggggaggaatg
	ACTB	5' junction	tggcctcgctgtccaccttc	tgcagcctgaaatgggctcatc
		3' junction	tcgcccttaattgtgagcggataac	gatgctcgctccaaccgactg
CRIS-PITCh in human cells	*FBL*	5' junction	acaccaagacagacatctctgtcccttg	atccgtatccaatgtggggaac
		3' junction	ccgcaacctccccttctacgag	tcagcaggtcaaggggaggaatg
TAL-PITCh in *B. mori*	*BLOS2*	5' junction	agcaaattgttgcaagggac	ccgctcgagttaattcacacaaaatgac
		3' junction	ataacgaccgcgtgagtcaa	agctagccacagtgtttatc
TAL-PITCh in *X. laevis*	*no29*	5' junction	agcataggcatctccctgatgtggt	ggtcttcatcatcactggctacaacg
		3' junction	gaaaggcggacaggtatccggtaag	ggcttccaaaagctgcctgtctcta

	fgk	5' junction	gtcaagaccattgttgaagaagt	tcagcttgccgtaggtggcatcgc
		3' junction	ggcgcgtcccattcgccatt	agacctcaagactccaaggcaacg
Southern blotting	*ACTB*	Outer probe	cgtgttctttgcactttctgcatgtc	tctccttaatgtcacgcacgatttcc
		mNG probe	gcaccggcaatcccaatgac	gtttgccgtttccggtggtg

Supplementary Table 7. Primers for PCR at the off-target sites.

	Locus	Forward primer (5′->3′)	Reverse primer (5′->3′)
TAL-PITCh at the *FBL* locus in HEK293T cells	TAL-OT1	cgtgctgggagggaagtctgt	ggtcaggcctgaagtcgaggt
	TAL-OT2	ggcgaccaccttgcttaaagcag	gccactgcacccggccatttt
	TAL-OT3	ctcgcctctgcctgcacactac	atgcgcctgtggtctcagctactc
	TAL-OT4	tgtccctgctaactgctcctg	tgggcaaaaagagcagaactc
	TAL-OT5	cggcaatgtgttttgggccc	cagcagactctgggggaggat
	TAL-OT6	atgatggtttccagcttcatcc	gaagcagatcctgtccaaaacc
CRIS-PITCh at the *FBL* locus in HEK293T cells	CRIS-OT1A	cttctgaatcctccttccatcc	ttgattctaggccaattttgagg
	CRIS-OT2A	atgtcatgcaggattggaagac	tataagccaaacaaggcttcctg
	CRIS-OT3A	gccatctcctaactgccaatatg	atcatacagccactgagtccaca
	CRIS-OT1B	atggctgttgatctgagagtcc	ttctcttggaaaaatgcctgtg
	CRIS-OT2B	agctctgcacctaagcctatcc	gtgggggaagacctactctgtg
	CRIS-OT3B	tagggtgatcagatggcttgag	atcctgagtcgcggaaaaatac
	CRIS-OT1C	gcacatggactgtgagagtcag	tatgctgggaatggagatgaac
	CRIS-OT2C	gctttgtaaagcctccctcttg	tggtctggcctgatttagtctg
	CRIS-OT3C	cactcaccactctgtgtcttcg	acacatcagggtgaccacacac
